# Trends in Bacterial Pathogens of Bats: Global Distribution and Knowledge Gaps

**DOI:** 10.1155/2023/9285855

**Published:** 2023-03-27

**Authors:** Tamara Szentivanyi, Clifton McKee, Gareth Jones, Jeffrey T. Foster

**Affiliations:** ^1^Pathogen and Microbiome Institute, Northern Arizona University, Flagstaff, AZ, USA; ^2^Centre for Ecological Research, Institute of Ecology and Botany, Vácrátót, Hungary; ^3^Department of Epidemiology, Johns Hopkins Bloomberg School of Public Health, Baltimore, MD, USA; ^4^School of Biological Sciences, University of Bristol, Bristol, UK

## Abstract

Bats have received considerable recent attention for infectious disease research because of their potential to host and transmit viruses, including Ebola, Hendra, Nipah, and multiple coronaviruses. These pathogens are occasionally transmitted from bats to wildlife, livestock, and to humans, directly or through other bridging (intermediate) hosts. Due to their public health relevance, zoonotic viruses are a primary focus of research attention. In contrast, other emerging pathogens of bats, such as bacteria, are vastly understudied despite their ubiquity and diversity. Here, we describe the currently known host ranges and geographic distributional patterns of potentially zoonotic bacterial genera in bats, using published presence-absence data of pathogen occurrence. We identify apparent gaps in our understanding of the distribution of these pathogens on a global scale. The most frequently detected bacterial genera in bats are *Bartonella*, *Leptospira*, and *Mycoplasma*. However, a wide variety of other potentially zoonotic bacterial genera are also occasionally found in bats, such as *Anaplasma*, *Brucella*, *Borrelia*, *Coxiella*, *Ehrlichia*, *Francisella*, *Neorickettsia*, and *Rickettsia*. The bat families Phyllostomidae, Vespertilionidae, and Pteropodidae are most frequently reported as hosts of bacterial pathogens; however, the presence of at least one bacterial genus was confirmed in all 15 bat families tested. On a spatial scale, molecular diagnostics of samples from 58 countries and four overseas departments and island states (French Guiana, Mayotte, New Caledonia, and Réunion Island) reported testing for at least one bacterial pathogen in bats. We also identified geographical areas that have been mostly neglected during bacterial pathogen research in bats, such as the Afrotropical region and Southern Asia. Current knowledge on the distribution of potentially zoonotic bacterial genera in bats is strongly biased by research effort towards certain taxonomic groups and geographic regions. Identifying these biases can guide future surveillance efforts, contributing to a better understanding of the ecoepidemiology of zoonotic pathogens in bats.

## 1. Introduction

In the past two decades, field studies have focused primarily on the diversity and distribution of medically important, bat-associated emerging viral pathogens, including Ebola, Hendra, Nipah, Marburg, and multiple coronaviruses [1–6]. Bats are certainly important reservoirs of myriad viruses [7], but their special role as hosts of zoonotic viruses when compared to other mammal taxa is a matter of debate. While some studies claim that bats host more zoonotic viruses per species when compared to other mammalian orders, such as rodents [8, 9], more recent work has shown that the number of zoonotic viruses found in bats is largely a function of host species diversity and that the proportion of zoonotic viruses varies little across mammalian orders [10]. Within bats, viral richness and the probability of viral spillover vary according to host and pathogen traits, including the geographic distribution of host species [8, 11, 12], virus taxa [10], the intersection of bat behavior and viral pathology [13], and host population genetic structure [14].

**Figure 1:**
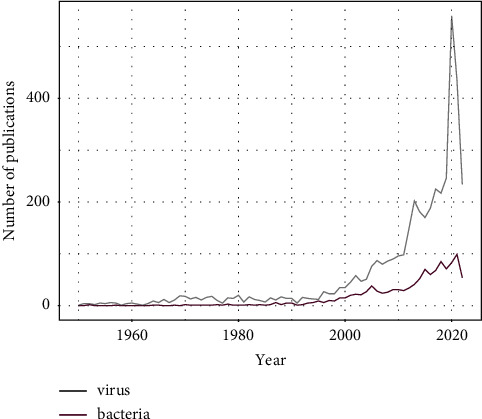
Number of studies focusing on bat-associated viruses (grey), and bacterial pathogens (purple) in PubMed 1948–2022 (October).

While viruses are of great concern for public health and host conservation due to potential spillover into susceptible species [15], persecution of bats, e.g., culling colonies due to fear of diseases [16], and disease-induced mass mortality [17–19], our knowledge on infectious bacterial pathogens of bats is limited. A strong study bias currently exists towards viral research compared with bacterial research in bats (Figure 1). Several potentially zoonotic bacterial pathogens have been detected in either bats or in their ectoparasites worldwide [20–24], and some evidence of bat-to-human transmission of bacterial pathogens has been documented [25–28]. For instance, bat-associated* Bartonella* sp. antibodies have been observed in humans in Africa, suggesting potential for inducing disease [25]. Additionally, human-pathogenic* Bartonella* sp. and *Borrelia* sp. have also been detected in bats and their ectoparasites, suggesting circulation in bat populations [27–29]. Moreover, genetically highly similar strains of *Mycoplasma* causing disease in humans have been found in bats and their associated ectoparasites [26].

Nevertheless, many such transmission events likely go undocumented due to a lack of sufficient surveillance effort. Bat-associated pathogens can be transmitted to humans, wildlife, and to domestic species through several transmission routes. These include exposure to body fluids (e.g., saliva, feces, blood) and arthropod vectors (e.g., ticks, fleas, mosquitoes) [30]. Additionally, humans can be exposed to bats and bat-associated pathogens through tourism and religious rituals (such as visiting caves) [31], bat hunting, and consumption [32], or by consuming food products contaminated with body fluids of infected animals (e.g., fruits eaten by fruit bats under roosts) [33, 34].

Bat-associated bacterial pathogens can be divided into multiple groups based on transmission routes: vector-borne ones (transmitted by hematophagous arthropods), such as *Anaplasma*, *Bartonella*, *Borrelia*, *Ehrlichia*, and *Rickettsia* spp.; directly transmitted pathogens (transmitted by close contact or contact with contaminated products), such as *Brucella* spp.; and pathogens transmitted either or both environmentally and via vectors (currently uncertain in bats), such as *Coxiella burnetii*, *Leptospira* spp., and *Mycoplasma* species.

In recent years, several newly and re-emerging infectious food- and waterborne bacterial pathogens have also been identified in bats, such as *Leptospira* spp. [22, 35, 36] and the recently discovered novel bat-associated* Brucella* sp. [37]. Some of these are recognized as potentially zoonotic and may have major consequences for human health (Suppl.  Table 1). For instance, annual reports of leptospirosis reach over one million human cases, resulting in about 58,900 deaths [38]. Brucellosis is one of the world's most common zoonotic disease, and the novel strain from bats from the Republic of Georgia is closely related to rodent strains found in the same region starting in the 1960s ([39]; J. Foster unpubl. data). Pathogenicity of the bat strain is currently unknown, yet the rodent strain is highly pathogenic to humans (A. Whatmore, unpubl. data). The pathology of bacterial diseases in bats is largely unknown [20].

Despite an increasing number of studies showing the frequent presence of bacterial pathogens in bats (Figure 1) and in their ectoparasites (which may serve as vectors), our understanding of the distribution and occurrence of these pathogens and the disease exposure of humans, wildlife, and domestic species is strikingly limited. Here, we aim to summarize the current knowledge on bacterial pathogen distributions and diversity both across host families and geographically, using known pathogen occurrence data in bats. Moreover, our goal was to identify underrepresented sampling regions and taxonomic groups, which can help develop more focused research projects, helping both bat conservation and public health research.

## 2. Materials and Methods

We collected bacterial pathogen presence-absence data from various literature sources published up to 2022 (last search October 2022). We included data resulting from molecular tests (PCR, qPCR) as well as data from microscopy, culture, and serological tests. For prevalence calculation, only those data where authors published individual presence-absence pathogen occurrence in their samples using molecular results were included in our dataset. Data were omitted when only positive data were published without information on total tested individuals and when data could not be referred to the individual level. In addition, only wild-caught or salvaged (collected postmortem) individuals were included in our dataset, excluding tests focusing on captive animals.

We focused on 11 bacterial genera with known zoonotic potential (Suppl.  Table 1): *Anaplasma*, *Bartonella*, *Borrelia*, *Brucella*, *Coxiella*, *Ehrlichia*, *Francisella*, *Leptospira*, *Mycoplasma*, *Neorickettsia*, and *Rickettsia*. Studies were retrieved from Google Scholar and PubMed using the following combinations of search words: *Chiroptera* or bat^*∗*^, *Anaplasma*^*∗*^, *Bartonella*^*∗*^, *Borrelia*^*∗*^, *Brucella*^*∗*^, *Coxiella*^*∗*^, *Ehrlichia*^*∗*^, *Francisella*^*∗*^, *Leptospira*^*∗*^, *Mycoplasma* OR *Haemoplasma*^*∗*^, *Neorickettsia*^*∗*^, *Rickettsia*^*∗*^, and bacterial pathogen. Additionally, following Birtles et al. [40], previous reports of *Grahamella* were collected and categorized as *Bartonella* in the dataset [40]. The PRISMA flowchart [41] documenting our systematic review can be found in the Supplementary Material (Suppl. Fig.  1). As we detail below, we excluded enteropathogenic bacteria that have been detected in bats, such as *Escherichia coli* and *Campylobacter*, *Salmonella*, and *Yersinia* species due to an incomplete understanding of their zoonotic potential.

For each dataset, we recorded detailed information whenever it was available, including surveillance method (culture, microscopy, serology, PCR, qPCR), sample source, host species, handling method (released, euthanized, salvaged), country, region, location, coordinates, collection date, number of individuals tested, and number of positive individuals.

For Figure 1, we searched the number of studies published and found in PubMed, using the search words: bat OR Chiroptera^*∗*^ AND bacteria^*∗*^, as well as bat OR Chiroptera^*∗*^ AND virus^*∗*^ to allow comparisons among the number of published papers on the different topics.

To compare estimates of bacterial pathogen prevalence, we focused only on estimates from studies that used molecular detection such as PCR or qPCR to limit issues with false negatives due to low sensitivity of alternative methods, including culture, microscopy, and microbiome sequencing. While data from these studies were excluded from prevalence comparisons, they were still used as presence measurements for the purpose of geographic and taxonomic analysis of bat hosts of bacterial pathogens. To assess whether bacterial pathogen prevalence varies across bat families, we performed a phylogenetic meta-analysis. We matched 295 bat host species of bacterial pathogens to a recent mammal phylogeny [42]. For 13 recently described species that could not be matched to the phylogeny, we matched the species to the congener with which they were most recently considered synonymous: *Eumops bonariensis* replaced *Eumops nanus*, *Molossus rufus* replaced *Molossus nigricans*, *Pteronotus davyi* replaced *Pteronotus fulvus*, *Pteronotus parnellii* replaced *Pteronotus mesoamericanus*, *Artibeus lituratus* replaced *Artibeus intermedius*, *Mimon crenulatum* replaced *Gardnerycteris keenani*, *Uroderma bilobatum* replaced *Uroderma convexum*, *Myotis keaysi* replaced *Myotis pilosatibialis*, *Chaerephon pumilus* replaced *Mops leucogaster* and *Mops pusillus*, *Miniopterus schreibersii* replaced *Miniopterus orianae*, *Myotis nigricans* replaced *Myotis caucensis*, and *Myotis brandtii* replaced *Myotis sibiricus*. Meta-analysis results, where these species were excluded instead of replacing, were qualitatively similar, so we only present the analysis with these replacements. We calculated the Freeman‒Tukey double arcsine transformed prevalence and the sampling variance for each host species and pathogen [43]. We then fit hierarchical meta-analysis models [44] with random effects for study, taxonomic species, and phylogeny (converting the bat phylogeny to a covariance matrix) to assess the heterogeneity in prevalence attributable to these factors.

We first fit a random effects model with only an intercept and used restricted maximum likelihood for unbiased estimation of the variance components for the random effects and derived *I*^2^ to quantify the percentage of variance in the meta-analysis that is attributable to true heterogeneity for each random effect [45]. We used Cochran's *Q* to test if the estimated heterogeneity in prevalence was greater than expected by the sampling error alone. We then fit a second mixed effects model for each pathogen that included the random effects and fixed effects for bat families to test for differences in prevalence among families. Pseudo-*R*^2^ was calculated based on the deviance explained by mixed effects model versus the model with only random effects [46]. Cochran's *Q* was also calculated on the mixed effects model to quantify whether the residual heterogeneity in prevalence was greater than expected after accounting for sampling error and fixed effects. Lastly, we back-transformed fitted coefficients (double arcsine transformed prevalence) and 95% confidence intervals from the mixed effects models to compare the meta-analysis estimated prevalence against untransformed prevalence, calculated by dividing total positive by total tested samples for each pathogen and bat family over all studies, with confidence intervals estimated using the Clopper‒Pearson exact method [47]. While meta-analysis was only performed on seven pathogen genera with sufficient testing (*n* > 1,000; including *Bartonella*, *Leptospira*, *Mycoplasma*, *Rickettsia*, *Anaplasma*, *Borrelia*, and *Coxiella*), estimated prevalence with exact confidence intervals was estimated by bat family for all 11 pathogen genera (Suppl. Tables 2–4).

Statistical analysis and data visualization were conducted in R 4.2.1 [48], using *ggplot2* [49], *ggpubr* [50], *leaflet* [51], and *rnaturalearth* packages [52]. We also used the *ape* [53], *metafor* [43], *ggtree* [54], *sp* [55], and *rgeos* [56] packages for manipulating and plotting phylogenies, meta-analysis, and mapping. Code for the meta-analysis was partly adapted from a recent preprint by Cohen et al. [57].

## 3. Results

**Figure 2:**
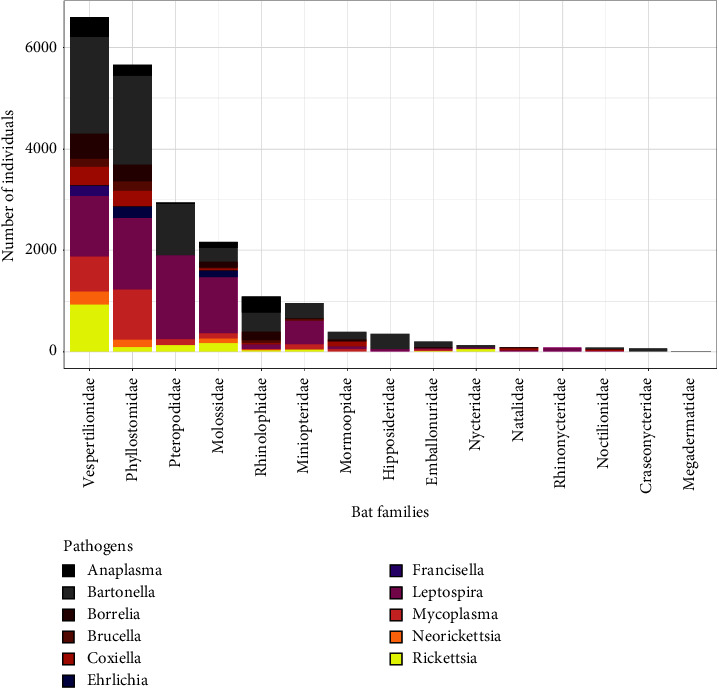
Number of tests (*n* = 22,910) performed (including presence and absence data) for the detection of 11 bacterial pathogenic genera across bat families. (Figure excludes data, when host identity was unreported).

We found a total of 23,412 pathogen presence-absence reports published in 152 studies, identifying 11 bacterial pathogen genera including *Bartonella* (*n* = 7,224), *Leptospira* (*n* = 7,032), *Mycoplasma* (*n* = 2,113), *Rickettsia* (*n* = 1,909), *Borrelia* (*n* = 1,508), *Anaplasma* (*n* = 1,064), *Coxiella* (*n* = 1,038), *Neorickettsia* (*n* = 494), *Brucella* (*n* = 473), *Ehrlichia* (*n* = 361), and *Francisella* (*n* = 196) (Figure 2, Suppl.  Table 5). Pathogen presence was screened in 319 identified bat species across 103 genera and 15 bat families. We provide data summaries below but caution about pervasive taxonomic and geographic sampling biases, which we account for in our analyses.

### 3.1. Distribution of Bat-Associated Bacterial Pathogens between Host Families

**Figure 3:**
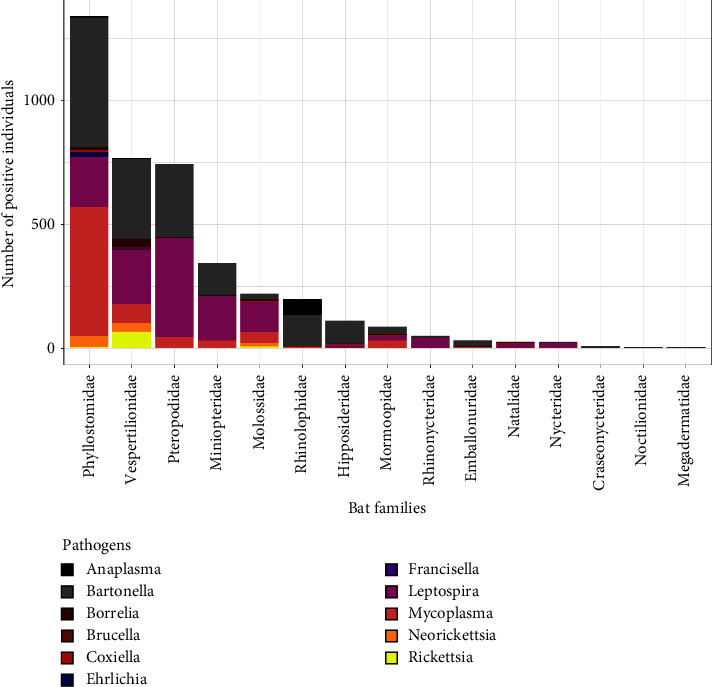
Distribution of positive tests (*n* = 4,314) for bat-associated bacterial pathogens across bat families and bacterial pathogen genera. (Figure excludes data, when host identity was unreported).

The occurrence of at least one bacterial pathogen genus was found in 15 bat families, across 77 genera and 215 species (Figure 3). A total of 4,351 positive tests were reported, including molecular screening, microscopy, and serology. The highest number of positive tests for at least one pathogen genus were reported from the families Phyllostomidae (*n* = 1,384), Vespertilionidae (*n* = 900), and Pteropodidae (*n* = 882). The highest diversity of pathogen genera was in the family Vespertilionidae, in which 11 bacterial genera were identified.

### 3.2. Detection Rate of Pathogens across Families

**Figure 4:**
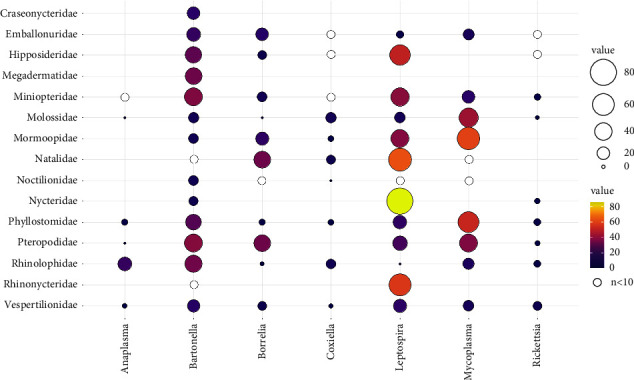
Bacterial pathogen detection rate (in %) of the seven most frequently screened pathogens across host families. White dots represent a sample number <10 individuals, and therefore were excluded from prevalence calculations. Only the results of molecular testing were included (i.e., PCR and qPCR).

To analyze detection rate, we only included the seven most frequently tested pathogens (*n* > 1,000). Limited data are available on *Anaplasma* infection in bats, but these data show the highest detection rate was in the family Rhinolophidae (21% occurrence in tested samples). *Bartonella* showed a relatively high detection rate in tested families, ranging from 7.3% in Nycteridae to 37.4% in Miniopteridae and 38.5% in Pteropodidae (Figure 4; Suppl. Table 2). The *Borrelia* detection rate ranged from 0% in the families Molossidae and Noctilionidae to 33.3% in the families Natalidae and Pteropodidae. *Coxiella* was detected at the highest rate in the family Molossidae (9.8%), whereas detection was lowest (0%) in the families Noctilionidae, Rhinolophidae, and Emballonuridae. *Leptospira* detection rate was highest in the family Nycteridae (85.7%) and lowest in Rhinolophidae and Noctilionidae (0%). *Mycoplasma* showed a high detection rate in three families: Mormoopidae (60.8%), Phyllostomidae (53.1%), and Molossidae (43.4%). Lastly, *Rickettsia* was detected across several families, though at low infection rates, ranging from 0.6% to 6.7% in Molossidae and Vespertilionidae, respectively (Figure 4; Suppl. Table 2). The frequency of sampled species relative to the number of species within a bat family shows a wide range (excluding unsampled families), e.g., showing that the least frequently sampled families are Pteropodidae (6%) and Hipposideridae (5.5%) for *Bartonella* and *Leptospira*, respectively (Suppl. Figures 2–7). For the four other pathogen genera with less frequent testing (*Brucella*, *Ehrlichia*, *Francisella*, and *Neorickettsia*), prevalence was generally low (<10%) in most families, but *Neorickettsia* had moderately high prevalence (30.4%) in Phyllostomidae (Suppl. Table 3).

Using phylogenetic meta-analysis that included random effects for study, bat species, and phylogenetic covariance and bat family as a fixed effect, we examined the amount of variation in prevalence that exists across studies of the seven most studied bacterial pathogens. We observed a high amount of heterogeneity (*I*^2^ > 80%) in prevalence estimates for *Bartonella*, *Leptospira*, *Mycoplasma*, and *Borrelia* and low to moderate heterogeneity (*I*^2^ between 18–56%) in the less frequently tested *Rickettsia*, *Anaplasma*, and *Coxiella* (Suppl. Table 4). Heterogeneity in prevalence was driven most by differences between studies and from the bat phylogeny, with lower contributions from additional species effects, for *Bartonella*, *Leptospira*, *Anaplasma*, and *Coxiella*; differences between studies and additional species effects were greater contributors to heterogeneity than the bat phylogeny for *Mycoplasma*, *Rickettsia*, and *Borrelia* (Suppl. Table 4). For all pathogens except *Coxiella*, the Cochran's *Q* test for residual heterogeneity was significant (*p* < 0.05) for the models with only random effects included, indicating that the sources of heterogeneity in pathogen prevalence were not sufficiently explained with the random effects. We included bat family as a fixed effect in the meta-analysis along with random effects and found that family explained some of the variance in prevalence for all pathogens except *Anaplasma*, with pseudo-*R*^2^ ranging from 11% for *Coxiella* to 70% for *Bartonella* (Suppl. Table 4). However, Cochran's *Q* test for the family fixed effect was not significant (*p* ≥ 0.05) for all seven pathogens, indicating that significant differences in prevalence among bat families could not be detected. Estimated prevalence from meta-analysis, accounting for random effects and differences among families, generally confirmed the bat families with the highest prevalence. Following meta-analysis, among the families with at least 10 samples tested, Miniopteridae had the highest estimated prevalence for *Bartonella*, Nycteridae for *Leptospira*, Phyllostomidae for *Mycoplasma*, Vespertilionidae for *Rickettsia*, Rhinolophidae for *Anaplasma*, Pteropodidae for *Borrelia*, and Molossidae for *Coxiella*. However, the confidence intervals following meta-analysis were much wider than the Clopper‒Pearson exact intervals (Suppl. Table 2). Cochran's *Q* test for residual heterogeneity for the mixed effects model was significant for *Bartonella*, *Leptospira*, *Mycoplasma*, *Rickettsia*, and *Borrelia*, indicating high remaining heterogeneity in prevalence among studies and species within a given bat family (Suppl. Table 2).

### 3.3. Geographical Distribution of Bat-Associated Bacterial Pathogens

**Figure 5:**
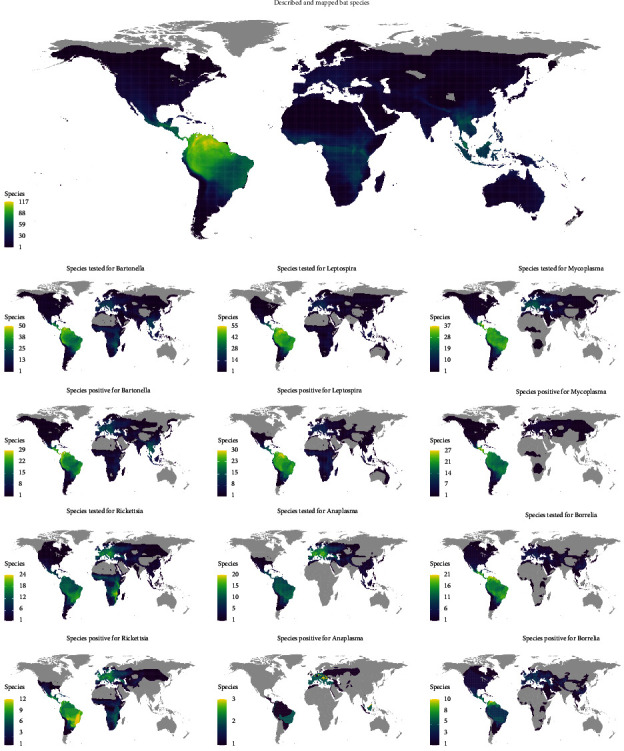
Geographical distribution of reported bat hosts of bacterial pathogens. Geographical ranges of reported bat host species for six widely tested bacterial pathogens (*Bartonella*, *Leptospira*, *Mycoplasma*, *Rickettsia*, *Anaplasma*, and *Borrelia*) were summarized from studies and data from the International Union for Conservation of Nature (IUCN). The plots display the number of bat species based on overlapping geographical ranges. The plots of bat species include 1,314 species with IUCN range data as of July 2022. Note the differences in scales of the diversity of bat species between maps of pathogens.

Samples from 58 countries and four overseas department and island states (French Guiana, Mayotte, New Caledonia, and Réunion) reported the molecular testing of at least one bacterial pathogen in bats. The highest number of tests for at least one bacterial pathogen genus were reported from Brazil (*n* = 3,578), China (*n* = 1,865), Hungary (*n* = 1,124), Madagascar (*n* = 1,092), and Peru (*n* = 987), whereas the most positive tests were reported from Brazil (*n* = 415), Belize (*n* = 371), Australia (*n* = 343), China (*n* = 336), and Peru (*n* = 324) (Suppl. Fig.  8). As for continents, the highest number of bat species were tested in South America (Figure 5). Sampling was unrepresentative in the case of several pathogens in certain geographical regions. For instance, Africa and Southeast Asia are largely neglected in bacterial pathogen research compared to other regions, except for *Bartonella* and *Leptospira* (Figure 5, Suppl. Figures 2–7).

### 3.4. Study Bias in Bat and Bacterial Pathogen Research

**Figure 6:**
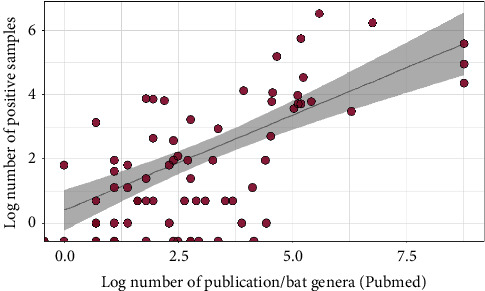
Effect of sampling effort on the number of positive samples across bat genera. Data show the relationship between the log number of positive tests per bat genus in relation to the log number of number of publications (PubMed) per bat genus in our dataset.

A Spearman rank correlation suggested a strong study bias, as the number of infected individuals positively correlated with the number of publications in PubMed (*n* = 104, df = 102, *p*=0.0001) (Figure 6).

## 4. Discussion

Wild animals serve as reservoirs of numerous potentially zoonotic pathogens, and bats represent no exception. Even though bacterial pathogens are ubiquitous in bats, limited information exists about their distribution, zoonotic potential, and their pathological effects on their hosts, particularly when compared to available information about bat-associated viral pathogens. During this work, we found that the most frequently detected bacterial pathogens in bats are *Bartonella*, *Leptospira*, and *Mycoplasma* species. These pathogens represent 88.5% of all reported bacterial pathogen occurrence across 15 bat families. Previous work has demonstrated that *Bartonella* spp. show a high diversity across bats in several host families [58]. Additionally, bats have played a key role in the radiation of mammal-associated* Bartonella* species and are suggested to be ancestral hosts of these pathogens [59]. *Bartonella* spp. are commonly detected not only in bats but also in their ectoparasites [21, 23, 60–63]. Although the vectors of *Bartonella* spp. are still unknown in bats, it has been suggested that ectoparasitic bat flies (Nycteribiidae, Streblidae) might be potential vectors [61, 64]. *Bartonella* spp. are indeed common in bats, but their ubiquity is in part due to a focus on this genus in surveillance studies.

*Leptospira* spp. are considered neglected but emerging infectious pathogens. Nevertheless, there are an increasing number of studies focusing on *Leptospira* occurrence in bats, showing it as common and widespread [65, 66]. In addition, *Leptospira* spp. also appear to be highly detectable across host families, based on the findings of the current work. Improved, targeted, and more widespread application of *Leptospira* spp. diagnostics will increase our understanding of the distribution and diversity of this genus in bats.

*Mycoplasma* spp. are widespread both geographically and across different mammalian orders [67], and are commonly observed among bats [68–70]. However, it generally shows a higher prevalence in vampire bats (Desmodontinae) compared with other mammalian groups [67]. Nevertheless, our understanding of *Mycoplasma* presence and diversity across bats is still limited, as about half of the known bat families have still not been targeted in *Mycoplasma* surveillance, and there is a large amount of heterogeneity in prevalence within and among sampled bat species that is not fully understood.

In addition, several other (partly or entirely) pathogenic bacterial taxa are largely unstudied in bats, such as *Borrelia*, *Francisella*, *Neorickettsia*, *Rickettsia*, *Brucella*, and vector-borne *Yersinia* species. Moreover, certain pathogenic taxa, such as *Chlamydiales* (e.g., *Chlamydia*-like organisms and *Waddlia* spp.), remain largely unexplored in bats. However, some limited occurrences have been reported in bats or in their ectoparasites [24, 68, 71–73]. A more complete understanding of these bacteria and potentially other species in bats requires additional focus and sampling including improved primer design and metagenomic sequencing.

Overall, strong sampling bias has been observed, both across different bat taxa and geographies. We currently have the most information about bacterial pathogen occurrence in the families Phyllostomidae, Pteropodidae, and Vespertilionidae, with the latter showing the highest diversity of observed pathogenic genera. Roughly, 72.5% of the pathogen surveillance data come from these three bat families, which represent about 66.9% of all known bat species. However, even within these families, surveillance data are reported from only about 22% of known bat species. The family Vespertilionidae also represents the highest species diversity compared to other bat families, with a worldwide distribution, which might partly explain high pathogen diversity within this family. Bat families with a small number of species and a restricted geographical distribution are generally underrepresented in bacterial pathogen surveillance studies, such as Megadermatidae, Natalidae, and Noctilionidae. Additionally, no bacterial pathogen surveillance effort has been reported for six further small bat families: Cistugidae, Furipteridae, Rhinopomatidae, Mystacinidae, Myzopodidae, and Thyropteridae (see Suppl. Figures 2–7). Knowledge on pathogen occurrence or diversity in these species is entirely lacking. Furthermore, little or no data have been reported from the Middle East, Central and South Asia, and from the majority of African countries, even though some of these areas host a high diversity of bats [74–76] (see Suppl. Figures 2–7).

Geographic bias was also prominent, with limited sampling for many bacterial pathogens in areas with the highest bat diversity. Most positive tests were observed from bats sampled in Africa; however, data were reported from only a handful of countries, with the majority still without any reported surveillance. Moreover, several pathogens have not been tested in bats from the continent (e.g., *Anaplasma*); therefore, conclusions regarding general prevalence cannot be drawn. In addition, more sampling is needed from understudied areas where bat diversity is generally high, such as Africa, Central and South Asia, and Southeast Asia, to reveal geographical patterns of pathogen diversity.

The phylogenetic meta-analysis that was performed for *Bartonella*, *Leptospira*, *Mycoplasma*, *Rickettsia*, *Borrelia*, and *Coxiella* also highlighted that much still needs to be done to understand the factors that contribute to heterogeneity in the prevalence of these pathogens across studies and bat taxonomy. While additional studies with greater coverage of bat species in different families and more testing of samples from species already covered in the database may help to more accurately assess heterogeneity in prevalence across bat species, there is also need for greater consistency and documentation of sampling approaches and testing methods. This will include more granular data on the seasonality of sampling, the timing of sampling relative to the breeding cycle of bat species, the exact location (with coordinates) of sampling sites, and their descriptions (e.g., cave versus tree roost). Some heterogeneity among studies is also partly attributable to methodological variance. For example, *Bartonella*, *Borrelia*, and *Mycoplasma* were tested in bat blood (or urine in the case of *Leptospira*) in some studies and in various tissues in other studies. Furthermore, a variety of PCR platforms including conventional PCR, nested PCR, and real-time PCR, and a diversity of gene targets were used to detect the same pathogen genus across studies, and these protocols vary in their sensitivity for pathogen detection. Improved documentation and greater consistency in detection methods [77, 78] could ameliorate some of the lingering issues with heterogeneity in prevalence among studies. Lastly, incorporation of species-level traits like body size, diet, fecundity, lifespan, roosting behavior, and sociality could help to explain some of the heterogeneity in prevalence among bat species and families and could improve future meta-analysis efforts [79].

### 4.1. Zoonotic Potential of Bat-Associated Bacterial Pathogens

Currently, we have limited understanding about the zoonotic potential of bat-borne bacterial pathogens. While there is some evidence of spillover of bat-associated* Bartonella* and *Borrelia* species, we lack sufficient surveillance to understand the scale of these events and whether spillovers of other pathogen genera occur. For instance, infection by *Candidatus* Bartonella mayotimonensis has been detected both in humans and bats, suggesting that bats are the reservoirs of this pathogen [28, 80–82]. Antibodies against *Bartonella rousetti* from Egyptian fruit bats (*Rousettus aegyptiacus*) were found in humans in Nigeria [25]. Also, high genetic similarity was found between *Bartonella* genotypes in European bats and bacteria detected in forest workers in Poland [83, 84]. Other vector-borne pathogens, such as *Borrelia johnsonii*, which was originally detected in the bat tick *Carios kelleyi* [85, 86], was later found in a human borreliosis patient also in the United States [87]. In another recently reported case, a patient in Zambia was hospitalized with high fever, days after visiting a cave and being bitten by a soft tick. Subsequent investigation found a novel relapsing fever *Borrelia* sp. in the patient and in soft ticks (*Ornithodoros faini*) and bats collected in the cave that the patient had visited [27]. Finally, *Leptospira* bacteria closely related to the human-pathogenic* L. interrogans* occur in bats [37, 88]. Clearly, a broad diversity of bat-associated bacterial pathogens are likely infecting humans, but the link to bats is often not recognized.

### 4.2. Transmission Routes of Vector-Borne Bacterial Pathogens in Bats

Transmission routes in vector-borne bat-associated bacterial pathogens remain largely unclear. These pathogens are often detected in ectoparasites of bats, such as *Anaplasma* and *Ehrlichia* in ticks, mites, and bat flies [21, 89–92]; *Bartonella* in bat flies, fleas, ticks, mites, and bat bugs [21, 23, 60, 63, 80, 85, 93, 104]; *Borrelia fainii*, *Borrelia johnsonii*, *Borrelia miyamotoi*, and other *Borrelia* spp. in ticks [29, 85, 86, 92, 105–107]; *Coxiella* in ticks [108]; *Mycoplasma* in bat flies and ticks [21, 26, 109]; and *Rickettsia* in bat flies, fleas, mites, and ticks [21, 85, 90, 92, 95, 104, 107, 110–118]. While the presence of potentially zoonotic bacterial pathogens in bat-associated ectoparasites is commonly detected, we still have little understanding about their role in pathogen transmission due to the lack of controlled experimental infection studies that show the potential of these vectors as competent hosts for transmission for any of these pathogens in bats [119, 120].

Additionally, pathogen surveillance data are not sufficient to reliably prove that the parasite is capable of transmission, as it is currently not possible to determine whether the presence of the bacteria in the residual bloodmeal is from an infected bat or the bacteria are truly replicating in the vectors. There may also be some genotypes or species within these pathogen genera that are present as endosymbionts of vectors (e.g., *Coxiella*, *Rickettsia*, and *Francisella*) that may not be transmissible to host bats. In addition, the transmission modes of certain pathogens (e.g., *Coxiella*, *Leptospira*, and *Mycoplasma* species) are currently unknown in bats. Ectoparasite and pathogen co-occurrence data may contribute to our understanding of parasite vectorial potential. A previous study showed that ectoparasite load and vector-borne *Bartonella* occurrence may be positively correlated in bats [82], although another study did not find this correlation [121]. Furthermore, other transmission modes, such as direct contact with body fluids (e.g., *Bartonella* is detectable in bat saliva and feces [121]), or vertical transmission of these pathogens (e.g., mother-to-offspring transmission of *Bartonella* in rodents [122, 123] or within ectoparasites [124]) are possible but still remain unexplored.

### 4.3. Ecological and Demographic Drivers of Pathogen Occurrence and Transmission

Only limited data are available that explore the drivers of bacterial pathogen occurrence and transmission in bats. Studies have showed that *Leptospira* infection in bats is associated with roost type, with higher prevalence in human-made structures [88], and is synchronized with the reproductive cycle of bats, showing an infection peak during late pregnancy and two months after the birth pulse [35]. Peaks in bat-associated virus shedding have also been shown to be affected by reproductive cycles [125]. Previous works have shown a higher *Bartonella* prevalence in large, male, and nonreproductive vampire bats, and in bats that feed on blood, when compared to bats with other diets [121, 126]. Furthermore, the *Bartonella* infection rate was higher in bat flies collected from bats roosting in caves rather than in buildings or trees [62, 104], suggesting that infection may be affected by roost type. Additionally, *Mycoplasma* prevalence was generally higher in vampire bats (compared to other bats and wildlife species), as well as in bat species with larger body sizes and larger colony sizes [67, 79]. More systematic collection of metadata on ecological and environmental covariates, bat host traits, the presence and behavior of ectoparasites, and bat health status (e.g., immunological parameters, body condition) is needed to understand predictors of bacterial infection in bats.

### 4.4. Pathogenicity of Bacterial Pathogens in Bats

Our understanding regarding the pathogenicity of bacterial pathogens in bats is still limited. We frequently operate under the widely accepted and often true assumption that bats experience no apparent disease from the pathogens they carry [127, 128]. Nevertheless, there are notable exceptions, such as in the cases of Tacaribe virus, Lloviu filovirus, and white-nose syndrome, which can cause individual or mass mortality in infected populations [17, 18, 129, 130]. Besides documented mass mortality events, assessments of bat health parameters in the field are not always recorded, and no widely accepted protocol exists. Therefore, it is challenging to determine whether there is consistent morbidity or mortality associated with bacterial infections in wild bat populations. Limited evidence is available about the pathogenicity of bacterial infections in bats, such as the case of fatal borreliosis in a bat found in the United Kingdom [131], or the mass mortality events associated with enteric *Yersinia* infection in both captive and wild bats [132, 133]. Postmortem examination of deceased individuals has shed light on the cause of death in some European bats [134], with systemic infections and septicemia caused by *Pasteurella multocida*, *Enterococcus faecalis*, *Enterococcus faecium*, and *Staphylococcus aereus* as a frequent clinical finding, often following traumatic injury. Another retrospective study on captive bats observed frequent bacterial infections affecting reproductive and haemolymphatic organs [135]. Development of better biomarkers of health status for bats in the field could challenge assumptions about the tolerance of bats for all types of infections and could aid in predicting when bacterial and viral shedding events might occur [136]. Overall, bacterial pathogens might contribute to significant morbidity and mortality in bats, both in captivity and in nature. However, our current knowledge is scarce about these processes and requires more attention.

### 4.5. Enteric Bacterial Pathogens of Bats

Although the main focus of this work was on nonenteric bat-associated bacterial pathogens, there has also been great development in the study of enteropathogens of bats. Enteropathogens are normally found in the gastrointestinal tract of the host and may potentially shed through body fluids and feces. Potentially zoonotic enteropathogenic bacteria have been detected in bats, such as *Escherichia coli* and *Campylobacter*, *Salmonella*, and *Yersinia* species [22, 137]. However, our knowledge on the scale of disease outbreaks and mortality by enteropathogens is scarce in nature, there is some evidence that they may cause mass mortality in bats, such as in the case of *Yersinia enterocolitica* [133]. Furthermore, *Y. pseudotuberculosis* may cause (mass) mortality in captive bats [132, 138, 139]. Generally, enteric *Yersinia* species are frequently detected in wild-caught bats and are suspected to be pathogenic to them based on histopathologic evidence [22, 134, 140].

*Escherichia coli* is commonly found in humans, and most strains are nonpathogenic [141]. However, some animal species, such as bats, host human-pathogenic strains in high diversity [142, 143], with emerging evidence indicating that some strains are antibiotic resistant [144–146], which may have veterinary or human medicine origins [147].

Some *Salmonella* and *Campylobacter* species may cause gastrointestinal disease both in animals and in humans. Several serotypes have been detected in bats, both in healthy and sick individuals [137, 142, 148, 149]. Furthermore, human-associated* Salmonella* and *Campylobacter* serotypes, such as *Salmonella typhi* and *Campylobacter jejuni* have been isolated from bats [137, 150, 151].

Overall, bat-associated enteropathogenic bacteria may pose a health risk to humans and domestic animals. Nevertheless, the scale of this risk is unknown under natural circumstances. Lastly, as bats possess a high diversity of pathogenic and nonpathogenic enteric bacteria, paired with bats' diverse ecological and physiological traits, they may be an ideal model system to understand mammal microbiome evolution, potentially contributing to public health advances [152].

### 4.6. Interactions between Pathogens

Ecological interactions between pathogens may be neutral, facultative (one infection increases the probability of another), or competitive (one infection decreases the probability of another). As an example, bacteria naturally occurring on bats have been shown to inhibit the growth of the pathogenic fungus *Pseudogymnoascus destructans* (causative agent of white-nose syndrome) [153]. Furthermore, *Leptospira* and paramyxovirus coinfections frequently occur in bats but without evidence of a directional interaction between the two pathogens [65]. Additionally, good evidence suggests multiple interactions of differing strength and direction (facultative vs. competitive) can occur in other wildlife systems [154, 155]. As we have little understanding of potential interactions between bacterial pathogens and other pathogens of bats, such as viruses, fungi, and ectoparasites, there are opportunities for future studies to address these questions.

### 4.7. Future Directions in Bacterial Pathogen Research in Bats

Identification of reservoir hosts using machine learning has been successfully carried out with high accuracy, such as identifying zoonotic pathogens of rodents [156] or bat reservoirs of filoviruses [157] and henipaviruses [158]. In addition, data-driven identification of vector species of a wide variety of zoonotic diseases has also been performed, evaluating the vectorial capacity of different mosquito and tick species [159, 160]. Usage of these predictive tools could help to prioritize sampling of bat species and their ectoparasites for surveillance of bacterial pathogens. Additionally, the development and increasing accessibility of accurate molecular epidemiological methods, such as using genomic and environmental DNA (eDNA) approaches, can contribute to bacterial pathogen surveillance on a larger geographic and taxonomic scale in bats [161–163]. Furthermore, using the combination of advanced molecular tools (e.g., metagenomic sequencing) and noninvasive collection methods (e.g., eDNA) could not only improve pathogen surveillance but also contribute to the conservation of the targeted species [63, 164, 165]. Additionally, building natural history collections can help the discovery of a many previously undescribed parasites and pathogens, contributing to the understanding of disease emergence and population dynamics over time [166, 167]. The collection of noninvasive samples should be prioritized to minimize existing threats towards threatened species [165]. Additionally, the development of technological tools such as microchips, PIT tags, and increasingly small GPS units to track individual bats could help us build knowledge on how frequently individuals are becoming infected (capture-recapture) and how animal movements and contact rates are contributing to transmission across space [168–170].

## 5. Conclusions

Several bat-associated bacterial pathogens have the potential to infect humans, likely due to close contact with infected bat hosts or with their ectoparasites. Bat-associated ectoparasites, such as ticks, can occasionally be found feeding on humans and other nonbat species. However, spillover events are seemingly rare or remain undocumented due to a lack of adequate surveillance. More information on pathogen occurrence, diversity, and seasonality is needed to successfully anticipate and prevent these events. Overall, there is still a lack of knowledge on several pathogenic bacterial taxa in bats and the reservoir potential of these animals. More targeted surveillance is urgently needed to better understand the ecoepidemiological role of bat-associated bacterial pathogens in disease maintenance and transmission. Lastly, pathogen diversity is likely connected to host diversity, therefore, improved sampling of those underrepresented areas and hosts are urgently needed. Increased access to research funding should be provided to researchers based in the Global South to ensure efficient and continuous surveillance of bacterial pathogens in underrepresented areas (e.g., sub-Saharan Africa, South and Southeast Asia, and South America), which could help to prevent the potential spillover of zoonotic bacterial pathogens, while prioritizing host conservation [171–174].

## Data Availability

Data supporting the conclusions of this work can be found in Supplementary Materials (Suppl. Figs. 1–8 and Suppl. Tables 1–5).

## Supplementary Materials

Supplementary Materials Supplementary Fig. 1: PRISMA flowchart diagram for systematic reviews indicating the pathogen screening process in publications [41]. Supplementary Fig. 2: geographical and taxonomic distribution of reported bat hosts of *Bartonella* bacteria. (A) Biogeographical patterns of bat families, sampling, and *Bartonella* host status. (B) Bat taxonomic diversity and *Bartonella* testing results. Data were compiled from field studies involving detection of *Bartonella* in wild bats. “Described” refers to the number of taxonomically described bat species per family based on the expert-curated Bat Species of the World database (Simmons and Cirranello, 2022). Supplementary Fig. 3: geographical and taxonomic distribution of reported bat hosts of *Leptospira* bacteria. (A) Biogeographical patterns of bat families, sampling, and *Leptospira* host status. (B) Bat taxonomic diversity and *Leptospira* testing results. Data were compiled from field studies involving detection of *Leptospira* in wild bats. “Described” refers to the number of taxonomically described bat species per family based on the expert-curated Bat Species of the World database (Simmons and Cirranello, 2022). Supplementary Fig. 4: geographical and taxonomic distribution of reported bat hosts of *Mycoplasma* bacteria. (A) Biogeographical patterns of bat families, sampling, and *Mycoplasma* host status. (B) Bat taxonomic diversity and *Mycoplasma* testing results. Data were compiled from field studies involving detection of *Mycoplasma* in wild bats. “Described” refers to the number of taxonomically described bat species per family based on the expert-curated Bat Species of the World database (Simmons and Cirranello, 2022). Supplementary Fig. 5: geographical and taxonomic distribution of reported bat hosts of *Rickettsia* bacteria. (A) Biogeographical patterns of bat families, sampling, and *Rickettsia* host status. (B) Bat taxonomic diversity and *Rickettsia* testing results. Data were compiled from field studies involving detection of *Rickettsia* in wild bats. “Described” refers to the number of taxonomically described bat species per family based on the expert-curated Bat Species of the World database (Simmons and Cirranello, 2022). Supplementary Fig. 6: geographical and taxonomic distribution of reported bat hosts of *Anaplasma* bacteria. (A) Biogeographical patterns of bat families, sampling, and *Anaplasma* host status. (B) Bat taxonomic diversity and *Anaplasma* testing results. Data were compiled from field studies involving detection of *Anaplasma* in wild bats. “Described” refers to the number of taxonomically described bat species per family based on the expert-curated Bat Species of the World database (Simmons and Cirranello, 2022). Supplementary Fig. 7: geographical and taxonomic distribution of reported bat hosts of *Borrelia* bacteria. (A) Biogeographical patterns of bat families, sampling, and *Borrelia* host status. (B) Bat taxonomic diversity and *Borrelia* testing results. Data were compiled from field studies involving detection of *Borrelia* in wild bats. “Described” refers to the number of taxonomically described bat species per family based on the expert-curated Bat Species of the World database (Simmons and Cirranello, 2022). Supplementary Fig. 8: geographical distribution of all tested bat individuals (A), and individuals that were positive for at least one pathogen (B) by country.
