# Supplementary material for: Trends in Bacterial Pathogens of Bats: Global Distribution and Knowledge Gaps
Source: Transbound Emerg Dis. 2023 Mar 27;2023:9285855. doi: 10.1155/2023/9285855 (PMC12017137; doi:10.1155/2023/9285855)
Supplement: Supplementary Materials — Supplementary Fig. 1: PRISMA flowchart diagram for systematic reviews indicating the pathogen screening process in publications [41]. Supplementary Fig. 2: geographical and taxonomic distribution of reported bat hosts of Bartonella bacteria. (A) Biogeographical patterns of bat families, sampling, and Bartonella host status. (B) Bat taxonomic diversity and Bartonella testing results. Data were compiled from field studies involving detection of Bartonella in wild bats. “Described” refers to the number of taxonomically described bat species per family based on the expert-curated Bat Species of the World database (Simmons and Cirranello, 2022). Supplementary Fig. 3: geographical and taxonomic distribution of reported bat hosts of Leptospira bacteria. (A) Biogeographical patterns of bat families, sampling, and Leptospira host status. (B) Bat taxonomic diversity and Leptospira testing results. Data were compiled from field studies involving detection of Leptospira in wild bats. “Described” refers to the number of taxonomically described bat species per family based on the expert-curated Bat Species of the World database (Simmons and Cirranello, 2022). Supplementary Fig. 4: geographical and taxonomic distribution of reported bat hosts of Mycoplasma bacteria. (A) Biogeographical patterns of bat families, sampling, and Mycoplasma host status. (B) Bat taxonomic diversity and Mycoplasma testing results. Data were compiled from field studies involving detection of Mycoplasma in wild bats. “Described” refers to the number of taxonomically described bat species per family based on the expert-curated Bat Species of the World database (Simmons and Cirranello, 2022). Supplementary Fig. 5: geographical and taxonomic distribution of reported bat hosts of Rickettsia bacteria. (A) Biogeographical patterns of bat families, sampling, and Rickettsia host status. (B) Bat taxonomic diversity and Rickettsia testing results. Data were compiled from field studies involving detection of Ricketts [file 9285855.f1.zip › SuppFigure3_Corrected.pdf]

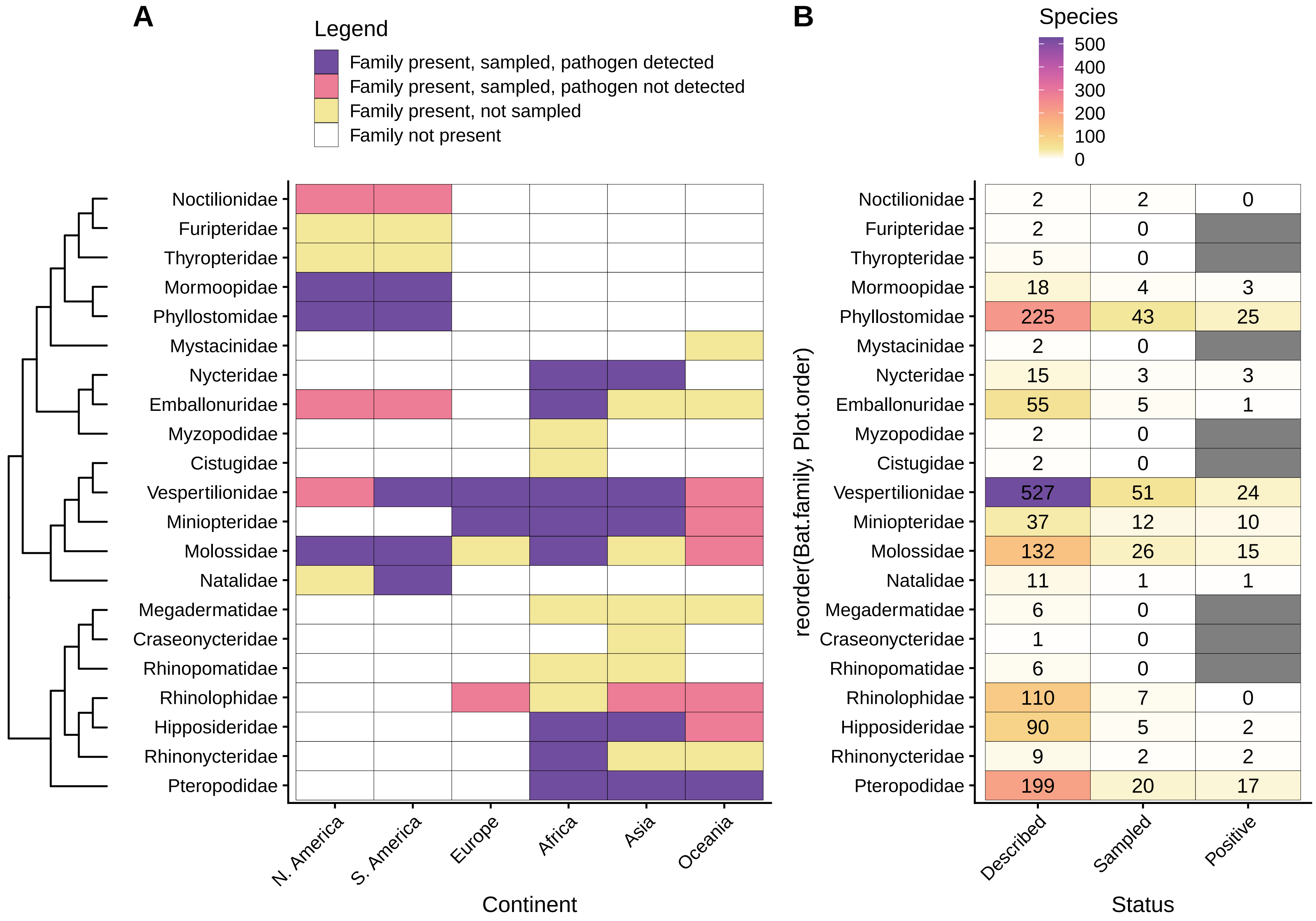
A
Legend
Family present, sampled, pathogen detected
Family present, sampled, pathogen not detected
Family present, not sampled
Family not present
Noctilionidae
Furipteridae
Thyropteridae
Mormoopidae
Phyllostomidae
Mystacinidae
Nycteridae
Emballonuridae
Myzopodidae
Cistugidae
Vespertilionidae
Miniopteridae
Molossidae
Natalidae
Megadermatidae
Craseonycteridae
Rhinopomatidae
Rhinolophidae
Hipposideridae
Rhinonycteridae
Pteropodidae
N. America
S. America
Europe
Africa
Asia
Oceania
Continent
B
Species
500
400
300
200
100
0
reorder(Bat.family, Plot.order)
Noctilionidae 2 2 0
Furipteridae 2 0
Thyropteridae 5 0
Mormoopidae 18 4 3
Phyllostomidae 225 43 25
Mystacinidae 2 0
Nycteridae 15 3 3
Emballonuridae 55 5 1
Myzopodidae 2 0
Cistugidae 2 0
Vespertilionidae 527 51 24
Miniopteridae 37 12 10
Molossidae 132 26 15
Natalidae 11 1 1
Megadermatidae 6 0
Craseonycteridae 1 0
Rhinopomatidae 6 0
Rhinolophidae 110 7 0
Hipposideridae 90 5 2
Rhinonycteridae 9 2 2
Pteropodidae 199 20 17
Described
Sampled
Positive
Status
