# Supplementary material for: Trends in Bacterial Pathogens of Bats: Global Distribution and Knowledge Gaps
Source: Transbound Emerg Dis. 2023 Mar 27;2023:9285855. doi: 10.1155/2023/9285855 (PMC12017137; doi:10.1155/2023/9285855)
Supplement: Supplementary Materials — Supplementary Fig. 1: PRISMA flowchart diagram for systematic reviews indicating the pathogen screening process in publications [41]. Supplementary Fig. 2: geographical and taxonomic distribution of reported bat hosts of Bartonella bacteria. (A) Biogeographical patterns of bat families, sampling, and Bartonella host status. (B) Bat taxonomic diversity and Bartonella testing results. Data were compiled from field studies involving detection of Bartonella in wild bats. “Described” refers to the number of taxonomically described bat species per family based on the expert-curated Bat Species of the World database (Simmons and Cirranello, 2022). Supplementary Fig. 3: geographical and taxonomic distribution of reported bat hosts of Leptospira bacteria. (A) Biogeographical patterns of bat families, sampling, and Leptospira host status. (B) Bat taxonomic diversity and Leptospira testing results. Data were compiled from field studies involving detection of Leptospira in wild bats. “Described” refers to the number of taxonomically described bat species per family based on the expert-curated Bat Species of the World database (Simmons and Cirranello, 2022). Supplementary Fig. 4: geographical and taxonomic distribution of reported bat hosts of Mycoplasma bacteria. (A) Biogeographical patterns of bat families, sampling, and Mycoplasma host status. (B) Bat taxonomic diversity and Mycoplasma testing results. Data were compiled from field studies involving detection of Mycoplasma in wild bats. “Described” refers to the number of taxonomically described bat species per family based on the expert-curated Bat Species of the World database (Simmons and Cirranello, 2022). Supplementary Fig. 5: geographical and taxonomic distribution of reported bat hosts of Rickettsia bacteria. (A) Biogeographical patterns of bat families, sampling, and Rickettsia host status. (B) Bat taxonomic diversity and Rickettsia testing results. Data were compiled from field studies involving detection of Ricketts [file 9285855.f1.zip › Supplementary_Table1-4_Corrected.docx]

*Supplementary Material*

*Supplementary Tables 1-4*

Trends in bacterial pathogens of bats: global distribution and knowledge gaps

Tamara Szentivanyi, Clifton McKee, Gareth Jones, Jeffrey T. Foster

**Supplementary Table 1.** Summary of major disease manifestations and symptoms observed in 11 focal bacterial pathogen genera. Where available, distinct signs and symptoms are described in humans, domesticated animals, and bats.

| **Bacterial genus** | **Disease manifestations** | | | |
| --- | --- | --- | --- | --- |
|  | **Humans** | **Domesticated animals** | **Bats** | **References** |
| *Anaplasma* | Human granulocytic anaplasmosis: fever, headache, chills, myalgia, nausea, vomiting, loss of appetite, diarrhea; severe illness may involve respiratory failure, organ failure, bleeding problems | Anaplasmosis (dogs, cats, horses, ruminants): fever, weight loss, diarrhea, aggressive behavior, decreased white blood cells, platelets in bloodstream, elevated liver enzymes, anemia, paleness or jaundice | Unknown | (Atif, 2015) |
| *Bartonella* | Cat scratch disease: fever, enlarged lymph nodes, papule/pustule at site of scratch; rare infections of eye, liver, spleen, brain, bones, heart valve (endocarditis)  Trench fever or five-day fever: fever, muscle and bone pain in the legs and back, skin lesions (bacillary angiomatosis), endocarditis  Carrion's disease: first phase (Orroya fever) includes fever, headache, muscle aches, abdominal pain, severe hemolytic anemia; later stage (verruga peruana) includes growths under the skin that develop into vascular sores, endocarditis | Bartonellosis in cats: often asymptomatic, but can rarely cause fever, vomiting, lethargy, red eyes, swollen lymph nodes, decreased appetite, endocarditis  Bartonellosis in dogs: fever, endocarditis and myocarditis, granulomatous lymphadenitis, cardiac arrhythmias, granulomatous rhinitis, and epistaxis  Bartonellosis in cattle: endocarditis | Unknown | (Chomel et al., 2009; Kaiser et al., 2011; Angelakis and Raoult, 2014) |
| *Borrelia* | Lyme disease: rash (*erythema migrans*) at site of tick bite, fatigue, headache, body aches, fever, chills; if untreated, may develop disseminated infection and neuroborreliosis or arthritis  Relapsing fever: fever, chills, headache, myalgia, arthralgia, nausea, vomiting | Lyme disease (dogs, cats, horses): chronic kidney disease, chronic joint disease  Relapsing fever (dogs, cats, birds, horses, cattle, pigs): fever, lethargy, ataxia, loss of appetite, weight loss, diarrhea | One case of fatal borreliosis in a wild bat in the UK | (Madigan, 1993; Evans et al., 2009; Cutler, 2010; Krupka and Straubinger, 2010; Wright et al., 2012; Shapiro, 2014; Elelu, 2018) |
| *Brucella* | Brucellosis: undulating fever, headache, arthralgia, night sweats, fatigue, loss of appetite, arthritis, spondylitis, endocarditis | Brucellosis (dogs, pigs, ruminants): abortion, stillbirth, mastitis, orchitis, epididymitis | Unknown | (Atluri et al., 2011; González-Espinoza et al., 2021) |
| *Coxiella* | Q fever: fever, headache, chills, pneumonia, hepatitis, endocarditis, abortion | Coxiellosis (dogs, cats, ruminants, poultry, pigeons): metritis, abortion, stillbirth, pneumonia | Unknown | (Arricau-Bouvery and Rodolakis, 2005; Kazar, 2005) |
| *Ehrlichia* | Human ewingii ehrlichiosis: fever, chills, headache, myalgia, malaise, thrombocytopenia, leukopenia  Human monocytotropic ehrlichiosis: fever, headache, malaise, myalgia, thrombocytopenia, leukopenia, elevated liver enzymes | Ehrlichiosis (dogs, cats): fever, lethargy, weight loss, lymphadenopathy, edema  Heartwater (ruminants): fever, loss of appetite, tremors, head pressing, cough, nasal discharge, tachycardia, edema | Unknown | (Buller et al., 1999; Peter et al., 2002; Ismail et al., 2010; Little, 2010) |
| *Francisella* | Tularemia: fever, skin ulcers, enlarged lymph nodes, pneumonia | Tularemia (cats, dogs, sheep): fever, lethargy, loss of appetite, enlarged lymph nodes  Francisellosis (farmed cod): loss of appetite, reduced swimming performance, dark pigmentation; swollen spleen, kidney and heart | Unknown | (Burroughs et al., 1945; Feldman, 2003; Nylund et al., 2006; Ottem et al., 2008) |
| *Leptospira* | Leptospirosis: headache, myalgia, fever, meningitis, liver failure, jaundice, pulmonary hemorrhage | Leptospirosis (dogs, horses, ruminants, pigs): loss of appetite, vomiting, lethargy, diarrhea, jaundice, dehydration | Unknown | (van de Maele et al., 2008; Verma et al., 2013; Ellis, 2015; Lane and Dore, 2016; Karpagam and Ganesh, 2020) |
| *Mycoplasma* | Hemoplasmosis: fever, splenomegaly, weight loss, asthenia, hepatomegaly, hemolytic anemia | Hemoplasmosis (cats, dogs, pigs, sheep): hemolytic anemia, depression, lethargy, weight loss, loss of appetite, fever | Unknown | (Messick, 2004; Hoelzle, 2008; Hattori et al., 2020; Descloux et al., 2021) |
| *Neorickettsia* | Sennetsu fever: fever, headache, myalgia, arthralgia | Potomac horse fever: fever, depression, diarrhea, loss of appetite, edema, abortion  Salmon poisoning disease (dogs): vomiting, diarrhea, loss of appetite, depression, fever, enlarged lymph nodes | Unknown | (Mulville, 1991; Headley et al., 2011) |
| *Rickettsia* | Epidemic typhus: fever, chills, headache, confusion, cough, myalgia, rash, nausea, vomiting, myocarditis, endocarditis  Murine typhus: headache, fever, myalgia, arthralgia, nausea, vomiting, rash, confusion  African tick bite fever: fever, headache, myalgia, eschar, lymphadenopathy, maculopapular rash  Rocky Mountain spotted fever: fever, nausea, vomiting, headache, myalgia, arthralgia malaise, maculopapular rash, petechial rash | Rocky Mountain spotted fever (dogs): fever, anorexia, lethargy, thrombocytopenia, petechial hemorrhage, neurological signs  Murine typhus (cats): asymptomatic or subclinical | Unknown | (Jensenius et al., 2003; Allison and Little, 2013; L. Li and Li, 2015; Biggs et al., 2016) |

**Supplementary Table 2.** Estimated prevalence across bat families for seven bacterial pathogens with sufficient testing (n > 1,000). Calculated prevalence values were estimated by dividing the total number of bats positive by the number tested, with confidence intervals estimated using the Clopper-Pearson exact method (Clopper and Pearson, 1934). These calculations included all samples identified to the family level, even if a bat was not identified to the species level. Meta-analysis prevalence was estimated using a phylogenetic multi-level mixed effects model (Viechtbauer, 2010) that included random effects for individual study, bat species, and phylogenetic covariance among bat species, and a fixed effect for bat family. Estimated confidence intervals reflect the heterogeneity in prevalence due to these factors. Note that in the meta-analysis, tested samples (and positives) from bats without species identification had to be dropped because they could not be matched with the bat phylogeny. “NA” indicates that either the bat family was not tested for a given pathogen or that the meta-analysis could not be performed after all the unidentified species in a family were excluded.

| **Bat family** | **Bacterial genus** | | | | | | |
| --- | --- | --- | --- | --- | --- | --- | --- |
|  | ***Bartonella*** | ***Leptospira*** | ***Mycoplasma*** | ***Rickettsia*** | ***Anaplasma*** | ***Borrelia*** | ***Coxiella*** |
| Craseonycteridae | Calculated prevalence: 10/57 (17.5 [8.7, 29.9]), Meta-analysis prevalence: 10/57 (18.9 [0, 66.7]) | NA | NA | NA | NA | NA | NA |
| Emballonuridae | Calculated prevalence: 16/78 (20.5 [12.2, 31.2]), Meta-analysis prevalence: 16/78 (20.9 [0.1, 60.4]) | Calculated prevalence: 1/23 (4.3 [0.1, 21.9]), Meta-analysis prevalence: 1/23 (11.7 [0, 52.7]) | Calculated prevalence: 4/35 (11.4 [3.2, 26.7]), Meta-analysis prevalence: 4/35 (6 [0, 54.3]) | Calculated prevalence: 0/6 (0 [0, 45.9]), Meta-analysis prevalence: 0/6 (2 [0, 37.7]) | NA | Calculated prevalence: 2/12 (16.7 [2.1, 48.4]), Meta-analysis prevalence: 2/12 (9.2 [0, 45.1]) | Calculated prevalence: 0/3 (0 [0, 70.8]), meta-analysis prevalence: 0/3 (16.5 [0, 75.1]) |
| Hipposideridae | Calculated prevalence: 89/294 (30.3 [25.1, 35.9]), Meta-analysis prevalence: 89/293 (21.1 [0.1, 62.7]) | Calculated prevalence: 13/36 (36.1 [20.8, 53.8]), Meta-analysis prevalence: 13/36 (42.5 [4.5, 86.6]) | NA | Calculated prevalence: 0/4 (0 [0, 60.2]), Meta-analysis prevalence: 0/4 (0.2 [0, 46.1]) | NA | Calculated prevalence: 3/47 (6.4 [1.3, 17.5]), Meta-analysis prevalence: NA | NA |
| Megadermatidae | Calculated prevalence: 5/15 (33.3 [11.8, 61.6]), Meta-analysis prevalence: 5/15 (34.3 [0, 86.2]) | NA | NA | NA | NA | NA | NA |
| Miniopteridae | Calculated prevalence: 74/198 (37.4 [30.6, 44.5]), Meta-analysis prevalence: 74/193 (43.9 [7.1, 85.3]) | Calculated prevalence: 177/450 (39.3 [34.8, 44]), Meta-analysis prevalence: 177/450 (38 [5, 79.6]) | Calculated prevalence: 30/114 (26.3 [18.5, 35.4]), Meta-analysis prevalence: 30/114 (30.5 [0, 86.9]) | Calculated prevalence: 1/31 (3.2 [0.1, 16.7]), Meta-analysis prevalence: 1/26 (5.4 [0, 25.1]) | Calculated prevalence: 0/1 (0 [0, 97.5]), Meta-analysis prevalence: 0/1 (0 [0, 100]) | Calculated prevalence: 2/21 (9.5 [1.2, 30.4]), Meta-analysis prevalence: 0/9 (14 [0, 56.7]) | Calculated prevalence: 0/1 (0 [0, 97.5]), meta-analysis prevalence: 0/1 (0 [0, 99.9]) |
| Molossidae | Calculated prevalence: 25/259 (9.7 [6.3, 13.9]), Meta-analysis prevalence: 25/254 (7.8 [0, 42.6]) | Calculated prevalence: 113/1084 (10.4 [8.7, 12.4]), Meta-analysis prevalence: 112/1006 (20.5 [0.5, 58.2]) | Calculated prevalence: 46/106 (43.4 [33.8, 53.4]), Meta-analysis prevalence: 41/93 (31.6 [0.2, 81.4]) | Calculated prevalence: 1/154 (0.6 [0, 3.6]), Meta-analysis prevalence: 1/149 (1.9 [0, 10.9]) | Calculated prevalence: 0/127 (0 [0, 2.9]), Meta-analysis prevalence: 0/127 (0.7 [0, 7.1]) | Calculated prevalence: 0/122 (0 [0, 3]), Meta-analysis prevalence: 0/122 (2.6 [0, 18.4]) | Calculated prevalence: 5/51 (9.8 [3.3, 21.4]), meta-analysis prevalence: 5/51 (10.6 [1.9, 24.1]) |
| Mormoopidae | Calculated prevalence: 10/112 (8.9 [4.4, 15.8]), Meta-analysis prevalence: 10/112 (14.5 [0, 51.6]) | Calculated prevalence: 19/50 (38 [24.7, 52.8]), Meta-analysis prevalence: 19/50 (21.5 [0, 64]) | Calculated prevalence: 31/51 (60.8 [46.1, 74.2]), Meta-analysis prevalence: 31/51 (29.4 [0, 80.5]) | NA | NA | Calculated prevalence: 6/32 (18.8 [7.2, 36.4]), Meta-analysis prevalence: 6/32 (0.7 [0, 17.6]) | Calculated prevalence: 2/95 (2.1 [0.3, 7.4]), meta-analysis prevalence: 2/95 (4.1 [0.1, 12.4]) |
| Natalidae | Calculated prevalence: 1/1 (100 [2.5, 100]), Meta-analysis prevalence: 1/1 (100 [0, 100]) | Calculated prevalence: 16/25 (64 [42.5, 82]), Meta-analysis prevalence: 16/25 (44.9 [0.5, 95.9]) | Calculated prevalence: 1/2 (50 [1.3, 98.7]), Meta-analysis prevalence: 1/2 (51.3 [0, 100]) | NA | NA | Calculated prevalence: 4/12 (33.3 [9.9, 65.1]), Meta-analysis prevalence: 4/12 (0.1 [0, 25.9]) | Calculated prevalence: 3/42 (7.1 [1.5, 19.5]), meta-analysis prevalence: 3/42 (5.3 [0, 18.7]) |
| Noctilionidae | Calculated prevalence: 2/22 (9.1 [1.1, 29.2]), Meta-analysis prevalence: 2/22 (7.1 [0, 55.2]) | Calculated prevalence: 0/8 (0 [0, 36.9]), Meta-analysis prevalence: 0/8 (2.2 [0, 58]) | Calculated prevalence: 0/1 (0 [0, 97.5]), Meta-analysis prevalence: 0/1 (0 [0, 100]) | NA | NA | Calculated prevalence: 0/4 (0 [0, 60.2]), Meta-analysis prevalence: 0/4 (7.6 [0, 63.9]) | Calculated prevalence: 0/32 (0 [0, 10.9]), meta-analysis prevalence: 0/32 (2.8 [0, 15.4]) |
| Nycteridae | Calculated prevalence: 4/55 (7.3 [2, 17.6]), Meta-analysis prevalence: 4/55 (20 [0, 67.8]) | Calculated prevalence: 18/21 (85.7 [63.7, 97]), Meta-analysis prevalence: 18/21 (74.4 [22.9, 100]) | NA | Calculated prevalence: 1/55 (1.8 [0, 9.7]), Meta-analysis prevalence: 1/55 (2.5 [0, 15.8]) | NA | NA | NA |
| Phyllostomidae | Calculated prevalence: 366/1303 (28.1 [25.7, 30.6]), Meta-analysis prevalence: 366/1300 (26 [2.6, 62.3]) | Calculated prevalence: 180/926 (19.4 [16.9, 22.1]), Meta-analysis prevalence: 165/839 (35.9 [7.1, 72.1]) | Calculated prevalence: 521/981 (53.1 [49.9, 56.3]), Meta-analysis prevalence: 521/978 (38 [4.7, 80.2]) | Calculated prevalence: 3/84 (3.6 [0.7, 10.1]), Meta-analysis prevalence: 3/84 (4.6 [0, 19.7]) | Calculated prevalence: 6/222 (2.7 [1, 5.8]), Meta-analysis prevalence: 6/222 (2.2 [0, 9.2]) | Calculated prevalence: 9/322 (2.8 [1.3, 5.2]), Meta-analysis prevalence: 9/322 (2.7 [0, 17]) | Calculated prevalence: 7/313 (2.2 [0.9, 4.6]), meta-analysis prevalence: 7/312 (5 [1, 11.7]) |
| Pteropodidae | Calculated prevalence: 224/582 (38.5 [34.5, 42.6]), Meta-analysis prevalence: 202/541 (14.2 [0, 50.3]) | Calculated prevalence: 384/1601 (24 [21.9, 26.2]), Meta-analysis prevalence: 177/1315 (18.2 [0.3, 54]) | Calculated prevalence: 41/111 (36.9 [28, 46.6]), Meta-analysis prevalence: 16/40 (43.9 [0, 99.3]) | Calculated prevalence: 2/131 (1.5 [0.2, 5.4]), Meta-analysis prevalence: 2/110 (3.2 [0, 14.6]) | Calculated prevalence: 0/25 (0 [0, 13.7]), Meta-analysis prevalence: 0/4 (1.8 [0, 48.6]) | Calculated prevalence: 59/177 (33.3 [26.4, 40.8]), Meta-analysis prevalence: 59/177 (33.3 [0.4, 83.7]) | NA |
| Rhinolophidae | Calculated prevalence: 124/366 (33.9 [29, 39]), Meta-analysis prevalence: 116/351 (23 [0.3, 64.9]) | Calculated prevalence: 0/78 (0 [0, 4.6]), Meta-analysis prevalence: 0/78 (14.7 [0, 58]) | Calculated prevalence: 2/15 (13.3 [1.7, 40.5]), Meta-analysis prevalence: 0/9 (26.1 [0, 90]) | Calculated prevalence: 0/27 (0 [0, 12.8]), Meta-analysis prevalence: 0/26 (5.5 [0, 24]) | Calculated prevalence: 63/301 (20.9 [16.5, 26]), Meta-analysis prevalence: 63/301 (15.5 [5, 30.2]) | Calculated prevalence: 1/174 (0.6 [0, 3.2]), Meta-analysis prevalence: 1/173 (12.8 [0.5, 36.3]) | Calculated prevalence: 0/23 (0 [0, 14.8]), meta-analysis prevalence: 0/23 (2.1 [0, 15.9]) |
| Rhinonycteridae | NA | Calculated prevalence: 42/72 (58.3 [46.1, 69.8]), Meta-analysis prevalence: 42/72 (52.3 [9.8, 92.9]) | NA | NA | NA | NA | NA |

**Supplementary Table 3.** Estimated prevalence across bat families for four bacterial pathogens with limited testing (n ≤ 1,000). Calculated prevalence values were estimated by dividing the total number of bats positive by the number tested, with confidence intervals estimated using the Clopper-Pearson exact method (Clopper and Pearson, 1934). These calculations included all samples identified to the family level, even if a bat was not identified to the species level.

| **Bat family** | **Bacterial genus** | | | |
| --- | --- | --- | --- | --- |
|  | ***Brucella*** | ***Ehrlichia*** | ***Francisella*** | ***Neorickettsia*** |
| Craseonycteridae | NA | NA | NA | NA |
| Emballonuridae | NA | NA | NA | NA |
| Hipposideridae | NA | NA | NA | NA |
| Megadermatidae | NA | NA | NA | NA |
| Miniopteridae | Calculated prevalence: 2/27 (7.4 [0.9, 24.3]) | NA | Calculated prevalence: 0/1 (0 [0, 97.5]) | Calculated prevalence: 0/1 (0 [0, 97.5]) |
| Molossidae | Calculated prevalence: 0/2 (0 [0, 84.2]) | Calculated prevalence: 0/127 (0 [0, 2.9]) | NA | Calculated prevalence: 12/100 (12 [6.4, 20]) |
| Mormoopidae | NA | NA | NA | NA |
| Natalidae | NA | NA | NA | NA |
| Noctilionidae | NA | NA | NA | NA |
| Nycteridae | NA | NA | NA | NA |
| Phyllostomidae | Calculated prevalence: 0/178 (0 [0, 2.1]) | Calculated prevalence: 20/222 (9 [5.6, 13.6]) | NA | Calculated prevalence: 42/138 (30.4 [22.9, 38.8]) |
| Pteropodidae | NA | NA | NA | NA |
| Rhinolophidae | Calculated prevalence: 0/52 (0 [0, 6.8]) | NA | Calculated prevalence: 0/5 (0 [0, 52.2]) | Calculated prevalence: 0/5 (0 [0, 52.2]) |
| Rhinonycteridae | NA | NA | NA | NA |

**Supplementary Table 4.** Statistics from phylogenetic meta-analysis of bacterial pathogen prevalence in bats. Meta-analysis accounts for random effects for individual study, bat species, and phylogenetic covariance among bat species, and a fixed effect for bat family. Heterogeneity (*I^2^*) was measured for the model with only random effects to quantify the contribution of each random factor to true heterogeneity in prevalence.

|  | **Bacterial genus** | | | | | | |
| --- | --- | --- | --- | --- | --- | --- | --- |
|  | ***Bartonella*** | ***Leptospira*** | ***Mycoplasma*** | ***Rickettsia*** | ***Anaplasma*** | ***Borrelia*** | ***Coxiella*** |
| Total variance in prevalence | 0.062 | 0.126 | 0.115 | 0.008 | 0.014 | 0.033 | 0.012 |
| **Random effects (null) model** | | | | | | | |
| Test for residual heterogeneity | Q(df = 291) = 1472, p < .0001 | Q(df = 248) = 1715, p < .0001 | Q(df = 128) = 1044, p < .0001 | Q(df = 77) = 103, p < .0001 | Q(df = 47) = 143, p < .0001 | Q(df = 46) = 279, p < .0001 | Q(df = 76) = 68, p = 0.73 |
| Heterogeneity, *I^2^* (%) |  |  |  |  |  |  |  |
| Total | 83 | 92.1 | 84.5 | 43. | 55.8 | 86.8 | 18.6 |
| Study | 35.5 | 50.6 | 46.1 | 38.4 | 21.6 | 86.8 | 15.5 |
| Species | 3.9 | 14.6 | 30.9 | 4.8 | 0.002 | 0 | 0.02 |
| Phylogeny | 43.5 | 26.9 | 7.5 | 0.001 | 34.2 | 0 | 3.1 |
| Variance components (sigma) |  |  |  |  |  |  |  |
| Study | 0.1754 | 0.2828 | 0.2228 | 0.1 | 0.0783 | 0.2417 | 0.0633 |
| Species | 0.0583 | 0.152 | 0.1823 | 0.0352 | 0.0007 | 0.0005 | 0.0022 |
| Phylogeny | 0.194 | 0.2061 | 0.09 | 0.0006 | 0.0985 | 0.0002 | 0.0283 |
| **Mixed effects model** | | | | | | | |
| Test for residual heterogeneity | QE(df = 278) = 1232, p < .0001 | QE(df = 236) = 1346, p < .0001 | QE(df = 119) = 588, p < .0001 | QE(df = 69) = 92, p = 0.035 | QE(df = 42) = 44, p = 0.38 | QE(df = 37) = 121, p < .0001 | QE(df = 68) = 60, p = 0.75 |
| Test of moderators | QM(df = 13) = 7.9, p = 0.85 | QM(df = 12) = 11.3, p = 0.5 | QM(df = 9) = 2.7, p = 0.97 | QM(df = 8) = 1.3, p = 0.99 | QM(df = 5) = 7, p = 0.22 | QM(df = 9) = 5.6, p = 0.78 | QM(df = 8) = 7, p = 0.54 |
| Variance components (sigma) |  |  |  |  |  |  |  |
| Study | 0.178 | 0.277 | 0.229 | 0.097 | 0.059 | 0.271 | 0.081 |
| Species | 0.018 | 0.147 | 0.138 | 0.000 | 0.001 | 0.000 | 0.002 |
| Phylogeny | 0.235 | 0.216 | 0.265 | 0.086 | 0.074 | 0.001 | 0.037 |
| Pseudo-R^2^ (%) | 68.9 | 19.9 | 12.4 | 15.3 | -6 | 23.2 | 10.9 |

**References**

Allison, R.W., and S.E. Little, 2013: Diagnosis of rickettsial diseases in dogs and cats. *Veterinary Clinical Pathology* **42**, 127–144, DOI: 10.1111/vcp.12040.

Angelakis, E., and D. Raoult, 2014: Pathogenicity and treatment of *Bartonella* infections. *International Journal of Antimicrobial Agents* **44**, 16–25, DOI: 10.1016/j.ijantimicag.2014.04.006.

Arricau-Bouvery, N., and A. Rodolakis, 2005: Is Q Fever an emerging or re-emerging zoonosis? *Vet. Res.* **36**, 327–349, DOI: 10.1051/vetres:2005010.

Atif, F.A., 2015: *Anaplasma marginale* and *Anaplasma phagocytophilum*: Rickettsiales pathogens of veterinary and public health significance. *Parasitology Research* **114**, 3941–3957, DOI: 10.1007/s00436-015-4698-2.

Atluri, V.L., M.N. Xavier, M.F. de Jong, A.B. den Hartigh, and R.M. Tsolis, 2011: Interactions of the human pathogenic *Brucella* species with their hosts. *Annu. Rev. Microbiol.* **65**, 523–541, DOI: 10.1146/annurev-micro-090110-102905.

Biggs, H.M., C.B. Behravesh, K.K. Bradley, F.S. Dahlgren, N.A. Drexler, J.S. Dumler, S.M. Folk, C.Y. Kato, R.R. Lash, M.L. Levin, R.F. Massung, R.B. Nadelman, W.L. Nicholson, C.D. Paddock, B.S. Pritt, and M.S. Traeger, 2016: Diagnosis and management of tickborne rickettsial diseases: Rocky Mountain spotted fever and other spotted fever group rickettsioses, ehrlichioses, and anaplasmosis — United States: a practical guide for health care and public health professionals. *Morbidity and Mortality Weekly Report: Recommendations and Reports* **65**, 1–44.

Buller, R.S., M. Arens, S.P. Hmiel, C.D. Paddock, J.W. Sumner, Y. Rikihisa, A. Unver, M. Gaudreault-Keener, F.A. Manian, A.M. Liddell, N. Schmulewitz, and G.A. Storch, 1999: *Ehrlichia ewingii*, a newly recognized agent of human ehrlichiosis. *N Engl J Med* **341**, 148–155, DOI: 10.1056/NEJM199907153410303.

Burroughs, A.L., R. Holdenried, D.S. Longanecker, and K.F. Meyer, 1945: A field study of latent tularemia in rodents with a list of all known naturally infected vertebrates. *Journal of Infectious Diseases* **76**, 115–119, DOI: 10.1093/infdis/76.2.115.

Chomel, B.B., R. w. Kasten, C. Williams, A. c. Wey, J. b. Henn, R. Maggi, S. Carrasco, J. Mazet, H. j. Boulouis, R. Maillard, and E. b. Breitschwerdt, 2009: *Bartonella* endocarditis. *Annals of the New York Academy of Sciences* **1166**, 120–126, DOI: 10.1111/j.1749-6632.2009.04523.x.

Clopper, C.J., and E.S. Pearson, 1934: The use of confidence or fiducial limits illustrated in the case of the binomial. *Biometrika* **26**, 404, DOI: 10.2307/2331986.

Cutler, S. j., 2010: Relapsing fever – a forgotten disease revealed. *Journal of Applied Microbiology* **108**, 1115–1122, DOI: 10.1111/j.1365-2672.2009.04598.x.

Descloux, E., O. Mediannikov, A.-C. Gourinat, J. Colot, M. Chauvet, I. Mermoud, D. Desoutter, C. Cazorla, E. Klement-Frutos, L. Antonini, A. Levasseur, V. Bossi, B. Davoust, A. Merlet, M.-A. Goujart, M. Oedin, F. Brescia, S. Laumond, P.-E. Fournier, and D. Raoult, 2021: Flying fox hemolytic fever, description of a new zoonosis caused by *Candidatus* Mycoplasma haemohominis. *Clinical Infectious Diseases* **73**, e1445–e1453, DOI: 10.1093/cid/ciaa1648.

Elelu, N., 2018: Tick-borne relapsing fever as a potential veterinary medical problem. *Veterinary Medicine and Science* **4**, 271–279, DOI: 10.1002/vms3.108.

Ellis, W.A., 2015: Animal leptospirosis, pp. 99–137. In: Adler, Ben (ed), *Leptospira* and leptospirosis. Berlin, Heidelberg: Springer.

Evans, N.J., K. Bown, D. Timofte, V.R. Simpson, and R.J. Birtles, 2009: Fatal borreliosis in bat caused by relapsing fever spirochete, United Kingdom. *Emerg Infect Dis* **15**, 1331–1333, DOI: 10.3201/eid1508.090475.

Feldman, K.A., 2003: Tularemia. *Journal of the American Veterinary Medical Association* **222**, 725–730, DOI: 10.2460/javma.2003.222.725.

González-Espinoza, G., V. Arce-Gorvel, S. Mémet, and J.-P. Gorvel, 2021: *Brucella*: reservoirs and niches in animals and numans. *Pathogens* **10**, 186, DOI: 10.3390/pathogens10020186.

Hattori, N., M. Kuroda, H. Katano, T. Takuma, T. Ito, N. Arai, R. Yanai, T. Sekizuka, S. Ishii, Y. Miura, T. Tokunaga, H. Watanabe, N. Nomura, J. Eguchi, H. Hasegawa, T. Nakamaki, T. Wakita, and Y. Niki, 2020: *Candidatus* Mycoplasma haemohominis in human, Japan. *Emerg Infect Dis* **26**, 11–19, DOI: 10.3201/eid2601.190983.

Headley, S.A., D.G. Scorpio, O. Vidotto, and J. Stephen Dumler, 2011: *Neorickettsia helminthoeca* and salmon poisoning disease: a review. *The Veterinary Journal* **187**, 165–173, DOI: 10.1016/j.tvjl.2009.11.019.

Hoelzle, L.E., 2008: Haemotrophic mycoplasmas: Recent advances in *Mycoplasma suis*. *Veterinary Microbiology* **130**, 215–226, DOI: 10.1016/j.vetmic.2007.12.023.

Ismail, N., K.C. Bloch, and J.W. McBride, 2010: Human ehrlichiosis and anaplasmosis. *Clinics in Laboratory Medicine* **30**, 261–292, DOI: 10.1016/j.cll.2009.10.004.

Jensenius, M., P.-E. Fournier, P. Kelly, B. Myrvang, and D. Raoult, 2003: African tick bite fever. *Lancet Infect Dis* **3**, 557–564, DOI: 10.1016/s1473-3099(03)00739-4.

Kaiser, P.O., T. Riess, F. O’Rourke, D. Linke, and V.A.J. Kempf, 2011: *Bartonella* spp.: throwing light on uncommon human infections. *International Journal of Medical Microbiology* **301**, 7–15, DOI: 10.1016/j.ijmm.2010.06.004.

Karpagam, K.B., and B. Ganesh, 2020: Leptospirosis: a neglected tropical zoonotic infection of public health importance—an updated review. *Eur J Clin Microbiol Infect Dis* **39**, 835–846, DOI: 10.1007/s10096-019-03797-4.

Kazar, J., 2005: *Coxiella burnetii* Infection. *Annals of the New York Academy of Sciences* **1063**, 105–114, DOI: 10.1196/annals.1355.018.

Krupka, I., and R.K. Straubinger, 2010: Lyme borreliosis in dogs and cats: background, diagnosis, treatment and prevention of infections with *Borrelia burgdorferi* sensu stricto. *Veterinary Clinics of North America: Small Animal Practice* **40**, 1103–1119, DOI: 10.1016/j.cvsm.2010.07.011.

Lane, A.B., and M.M. Dore, 2016: Leptospirosis: a clinical review of evidence based diagnosis, treatment and prevention. *World Journal of Clinical Infectious Diseases* **6**, 61–66, DOI: 10.5495/wjcid.v6.i4.61.

Li, L., and G. Li, 2015: Epidemic and endemic typhus, pp. 89–94. In: Li, Hongjun (ed), Radiology of Infectious Diseases: Volume 2. Dordrecht: Springer Netherlands.

Little, S.E., 2010: Ehrlichiosis and anaplasmosis in dogs and cats. *Veterinary Clinics of North America: Small Animal Practice* **40**, 1121–1140, DOI: 10.1016/j.cvsm.2010.07.004.

Madigan, J.E., 1993: Lyme disease (Lyme borreliosis) in horses. *Veterinary Clinics of North America: Equine Practice* **9**, 429–434, DOI: 10.1016/S0749-0739(17)30409-1.

Messick, J.B., 2004: Hemotrophic mycoplasmas (hemoplasmas): a review and new insights into pathogenic potential. *Veterinary Clinical Pathology* **33**, 2–13, DOI: 10.1111/j.1939-165X.2004.tb00342.x.

Mulville, P., 1991: Equine monocytic ehrlichiosis (Potomac horse fever): a review. *Equine Veterinary Journal* **23**, 400–404, DOI: 10.1111/j.2042-3306.1991.tb03749.x.

Nylund, A., K.F. Ottem, K. Watanabe, E. Karlsbakk, and B. Krossøy, 2006: *Francisella* sp. (Family Francisellaceae) causing mortality in Norwegian cod (*Gadus morhua*) farming. *Arch Microbiol* **185**, 383–392, DOI: 10.1007/s00203-006-0109-5.

Ottem, K.F., A. Nylund, T.E. Isaksen, E. Karlsbakk, and Ø. Bergh, 2008: Occurrence of *Francisella piscicida* in farmed and wild Atlantic cod, *Gadus morhua* L., in Norway. *Journal of Fish Diseases* **31**, 525–534, DOI: 10.1111/j.1365-2761.2008.00930.x.

Page, M.J., J.E. McKenzie, P.M. Bossuyt, I. Boutron, T.C. Hoffmann, C.D. Mulrow, L. Shamseer, J.M. Tetzlaff, E.A. Akl, S.E. Brennan, R. Chou, J. Glanville, J.M. Grimshaw, A. Hróbjartsson, M.M. Lalu, T. Li, E.W. Loder, E. Mayo-Wilson, S. McDonald, L.A. McGuinness, L.A. Stewart, J. Thomas, A.C. Tricco, V.A. Welch, P. Whiting, and D. Moher, 2021: The PRISMA 2020 statement: an updated guideline for reporting systematic reviews. *Systematic Reviews* **10**, 1–11, DOI: 10.1186/s13643-021-01626-4.

Peter, T.F., M.J. Burridge, and S.M. Mahan, 2002: *Ehrlichia ruminantium* infection (heartwater) in wild animals. *Trends in Parasitology* **18**, 214–218, DOI: 10.1016/S1471-4922(02)02251-1.

Shapiro, E.D., 2014: Lyme disease. *N Engl J Med* **370**, 1724–1731, DOI: 10.1056/NEJMcp1314325.

Simmons, N.B., and A.L. Cirranello, 2022: Bat Species of the World: A taxonomic and geographic database [Online] Available at https://batnames.org/ (accessed May 8, 2022).

van de Maele, I., A. Claus, F. Haesebrouck, and S. Daminet, 2008: Leptospirosis in dogs: a review with emphasis on clinical aspects. *Veterinary Record* **163**, 409–413, DOI: 10.1136/vr.163.14.409.

Verma, A., B. Stevenson, and B. Adler, 2013: Leptospirosis in horses. *Veterinary Microbiology* **167**, 61–66, DOI: 10.1016/j.vetmic.2013.04.012.

Viechtbauer, W., 2010: Conducting meta-analyses in R with the metafor package. *Journal of Statistical Software* **36**, 1–48.

Wright, W.F., D.J. Riedel, R. Talwani, and B.L. Gilliam, 2012: Diagnosis and management of Lyme disease. *afp* **85**, 1086–1093.
