# Supplementary material for: Trends in Bacterial Pathogens of Bats: Global Distribution and Knowledge Gaps
Source: Transbound Emerg Dis. 2023 Mar 27;2023:9285855. doi: 10.1155/2023/9285855 (PMC12017137; doi:10.1155/2023/9285855)
Supplement: Supplementary Materials — Supplementary Fig. 1: PRISMA flowchart diagram for systematic reviews indicating the pathogen screening process in publications [41]. Supplementary Fig. 2: geographical and taxonomic distribution of reported bat hosts of Bartonella bacteria. (A) Biogeographical patterns of bat families, sampling, and Bartonella host status. (B) Bat taxonomic diversity and Bartonella testing results. Data were compiled from field studies involving detection of Bartonella in wild bats. “Described” refers to the number of taxonomically described bat species per family based on the expert-curated Bat Species of the World database (Simmons and Cirranello, 2022). Supplementary Fig. 3: geographical and taxonomic distribution of reported bat hosts of Leptospira bacteria. (A) Biogeographical patterns of bat families, sampling, and Leptospira host status. (B) Bat taxonomic diversity and Leptospira testing results. Data were compiled from field studies involving detection of Leptospira in wild bats. “Described” refers to the number of taxonomically described bat species per family based on the expert-curated Bat Species of the World database (Simmons and Cirranello, 2022). Supplementary Fig. 4: geographical and taxonomic distribution of reported bat hosts of Mycoplasma bacteria. (A) Biogeographical patterns of bat families, sampling, and Mycoplasma host status. (B) Bat taxonomic diversity and Mycoplasma testing results. Data were compiled from field studies involving detection of Mycoplasma in wild bats. “Described” refers to the number of taxonomically described bat species per family based on the expert-curated Bat Species of the World database (Simmons and Cirranello, 2022). Supplementary Fig. 5: geographical and taxonomic distribution of reported bat hosts of Rickettsia bacteria. (A) Biogeographical patterns of bat families, sampling, and Rickettsia host status. (B) Bat taxonomic diversity and Rickettsia testing results. Data were compiled from field studies involving detection of Ricketts [file 9285855.f1.zip › Supplementary_Table_5_Corrected.docx]

*Supplementary Material*

*Supplementary Table 5*

Trends in bacterial pathogens of bats: global distribution and knowledge gaps

Tamara Szentivanyi, Clifton McKee, Gareth Jones, Jeffrey T. Foster

**Supplementary Table 5.** Table indicating the number of tests/number of positive tests for each pathogenic group in the dataset. Additional details, such as country, bat family and number of publications for each pathogenic group is given.

*indicating countries/bat families with positive infection data

| **Pathogen** | **N total tested/N positive** | **Country (positive ind*)** | **Bat families tested/ positive*** | **Reference** | **Number of publications** |
| --- | --- | --- | --- | --- | --- |
| *Anaplasma* | 1064/76 | Argentina, Brazil*, China, Federation of Saint Christopher and Nevis, France*, Hungary, Laos, Malaysia*, Netherlands*, Poland* | Miniopteridae, Molossidae, Phyllostomidae*, Pteropodidae, Rhinolophidae*, Vespertilionidae* | (Cicuttin et al. 2013, 2017; Ferreira dos Santos 2013; Reeves et al. 2016; Afonso and Goydadin 2018; Han et al. 2018b; Hornok et al. 2018; Szubert-Kruszyńska et al. 2019; Ikeda et al. 2021; Mahyudin and Kumar 2022; Nawtaisong et al. 2022) | 11 |
| *Bartonella* | 7224/1732 | Algeria*, Argentina*, Austria, Belize*, Brazil*, Chile*, China*, Columbia*, Costa Rica*, Czech Republic, Federation of Saint Christopher and Nevis*, Finland*, France*, French Guiana*, Georgia*, Guatemala*, Hungary*, Italy*, Japan*, Kenya*, Madagascar*, Malaysia*, Mexico*, Netherlands*, Nigeria*, Peru*, Poland*, Puerto Rico*, Romania*, Slovenia*, South Africa*, Spain*, Swaziland (Eswatini)*, Taiwan*, Thailand*, United Kingdom*, United States*, Vietnam*, Zambia* | Craseonycteridae*, Emballonuridae*, Hipposideridae*, Megadermatidae*, Miniopteridae*, Molossidae*, Mormoopidae*, Natalidae*, Noctilionidae*, Nycteridae*, Phyllostomidae*, Pteropodidae*, Rhinonycteridae*, Rhinolophidae*, Vespertilionidae* | (Goedbloed et al. 1964; Sebek 1975; Gardner et al. 1987; Concannon et al. 2005; Reeves et al. 2006, 2016; Kosoy et al. 2010; Bai et al. 2011, 2012, 2017, 2018; Lin et al. 2012; Kamani et al. 2014; Veikkolainen et al. 2014; Anh et al. 2015; Judson et al. 2015; Lilley et al. 2015, 2017; Olival et al. 2015; Brook et al. 2015; Davoust et al. 2016; Dietrich et al. 2016, 2017; Leulmi et al. 2016; Wray et al. 2016; Cicuttin et al. 2017; Han et al. 2017, 2021; Ikeda et al. 2017, 2020; McKee et al. 2017, 2021; Stuckey et al. 2017a, b; Urushadze et al. 2017; Corduneanu et al. 2018, 2021; Ferreira et al. 2018; Hou et al. 2018; Raya et al. 2018; Vengust et al. 2018; Becker et al. 2018a; André et al. 2019; Szubert-Kruszyńska et al. 2019; Gonçalves-Oliveira et al. 2020; Müller et al. 2020; Nabeshima et al. 2020, 2022; Qiu et al. 2020; Sun et al. 2020; Szentiványi et al. 2020; Braga et al. 2020; Li et al. 2021a; Poofery et al. 2021; Mahyudin and Kumar 2022; Mitchell et al. 2022; Carvajal-Agudelo et al. 2022) | 57 |
| *Borrelia* | 1508/143 | Algeria, Argentina, Brazil, Canada*, China*, Colombia*, Mexico*, Russia*, Tunisia, United Kingdom*, United States*, Zambia* | Emballonuridae*, Hipposideridae*, Miniopteridae*, Molossidae, Mormoopidae*, Natalidae*, Noctilionidae, Phyllostomidae*, Pteropodidae*, Rhinolophidae*, Vespertilionidae* | (Marinkelle and Grose 1968; Hanson 1970; Evans et al. 2009; Leulmi et al. 2016; Cicuttin et al. 2017; Qiu et al. 2019; Sanseverino et al. 2019; Zabashta et al. 2019; Banerjee et al. 2020; Muñoz-Leal et al. 2020; Colunga-Salas et al. 2020; Han et al. 2020; Selmi et al. 2021; Li et al. 2021b; Carvajal-Agudelo et al. 2022) | 15 |
| *Brucella* | 473/12 | Brazil*, China*, Georgia* | Emballonuridae*, Hipposideridae*, Miniopteridae*, Molossidae, Phyllostomidae*, Pteropodidae*, Rhinolophidae, Vespertilionidae* | (Ricciardi et al. 1976; Bai et al. 2017; Imnadze et al. 2020; Sun et al. 2020; Ferreira et al. 2021) | 5 |
| *Coxiella* | 1038/33 | Algeria, Australia*, Brazil*, Chile*, China, Colombia*, French Guiana, Hungary, India*, Malaysia*, Netherlands*, Slovenia*, Tunisia | Emballonuridae, Miniopteridae, Molossidae*, Mormoopidae, Noctilionidae, Phyllostomidae*, Pteropodidae*, Rhinolophidae, Vespertilionidae* | (Yadav and Sethi 1980; Gardon et al. 2001; Davoust et al. 2014; Tozer et al. 2014; Leulmi et al. 2016; Ferreira et al. 2018; Vengust et al. 2018; Han et al. 2018b; Hornok et al. 2018; Müller et al. 2020; Selmi et al. 2021; Mahyudin and Kumar 2022; Silva-Ramos et al. 2022b) | 13 |
| *Ehrlichia* | 361/25 | Argentina, Brazil*, Columbia*, Federation of Saint Christopher and Nevis*, Tunisia* | Molossidae, Phyllostomidae*, Vespertilionidae* | (Cicuttin et al. 2013, 2017; Ferreira dos Santos 2013; Reeves et al. 2016; Ikeda et al. 2021; Selmi et al. 2021; Carvajal-Agudelo et al. 2022) | 7 |
| *Francisella* | 196/3 | Georgia, Hungary, Netherlands* | Miniopteridae, Rhinolophidae, Vespertilionidae* | (Hornok et al. 2018; Imnadze et al. 2020) | 2 |
| *Leptospira* | 7032/1372 | Argentina*, Armenia, Australia*, Azerbaijan*, Brazil*, China*, Colombia*, Comoros*, Czech Republic*, Denmark*, Ecuador, Georgia*, Grenada*, Laos*, Madagascar*, Malaysia*, Mauritius*, Mayotte (France)*, Mexico*, Mozambique*, New Caledonia*, Nigeria*, Peru*, Poland*, Reunion Island (France)*, Russia, South Africa*, Sudan*, Slovakia*, Swaziland (Eswatini)*, Tanzania*, United States, Zambia* | Emballonuridae*, Hipposideridae*, Miniopteridae*, Molossidae*, Mormoopidae*, Noctilionidae, Nycteridae*, Phyllostomidae*, Pteropodidae*, Rhinonycteridae*, Rhinolophidae, Vespertilionidae* | (Noguchi 1919; Emanuel et al. 1964; Tagi-Zade et al. 1970; Fennestad and Borg-Petersen 1972; Lins and Rosa 1976; Everard et al. 1983; Sebek et al. 1989; Bunnell et al. 2000; Smythe et al. 2002; Matthias et al. 2005; Cox et al. 2005; Zetun et al. 2009; Bessa et al. 2010; Tulsiani et al. 2011; Lagadec et al. 2012, 2016; Desvars et al. 2012, 2013; Thayaparan et al. 2013, 2015; Dietrich et al. 2017, 2018a, b; Harkin et al. 2014; Mgode et al. 2014; Ramirez et al. 2014; Dietrich et al. 2014, 2015; Ogawa et al. 2015; Gomard et al. 2016; Bai et al. 2017; Mayer et al. 2017; Ballados-González et al. 2018; Han et al. 2018a; Victoria et al. 2018; Mateus et al. 2019; Zhao et al. 2019; Bevans et al. 2020; Torres-Castro et al. 2020; Ferreira et al. 2021; Kamani et al. 2021; Monroy et al. 2021; Ramos-Nino et al. 2021; Saraullo et al. 2021; Seidlova et al. 2021, 2022; Nawtaisong et al. 2022; Silva-Ramos et al. 2022a; Soupé‐Gilbert et al. 2022) | 49 |
| *Mycoplasma* | 2113/755 | Australia*, Belize*, Brazil*, Chile*, China*, Costa Rica*, Germany*, Hungary, India*, Netherlands*, New Caledonia*, Nigeria*, Peru*, Spain*, Switzerland, Tunisia, United States* | Emballonuridae*, Miniopteridae*, Molossidae*, Mormoopidae*, Natalidae*, Noctilionidae, Phyllostomidae*, Pteropodidae*, Rhinolophidae*, Vespertilionidae* | (Mascarelli et al. 2014; Millán et al. 2015, 2019; Banskar et al. 2016; Ikeda et al. 2017, 2022; Volokhov et al. 2017; Becker et al. 2018b; Newman et al. 2018; Becker et al. 2020; Hornok et al. 2018; Yuan et al. 2019; Holz et al. 2019; Correia dos Santos et al. 2020; Di Cataldo et al. 2020; Fritschi et al. 2020; Selmi et al. 2021; Descloux et al. 2021; Collere et al. 2022) | 19 |
| *Neorickettsia* | 494/88 | Argentina*, Brazil*, Hungary, Netherlands*, United States* | Miniopteridae, Molossidae*, Phyllostomidae*, Rhinolophidae, Vespertilionidae* | (Pusterla et al. 2003; Gibson et al. 2005; Cicuttin et al. 2013, 2017; Ferreira dos Santos 2013; Hornok et al. 2018; Ikeda et al. 2021) | 7 |
| *Rickettsia* | 1909/122 | Algeria, Argentina*, Brazil*, Chile, China*, Costa Rica, Czech Republic*, Federation of Saint Christopher and Nevis*, Hungary*, Laos, Malaysia, Netherlands*, Poland, Romania*, Slovenia*, South Africa*, Swaziland (Eswatini)*, Tunisia | Emballonuridae, Hipposideridae, Miniopteridae*, Molossidae*, Nycteridae*, Phyllostomidae*, Pteropodidae*, Rhinolophidae*, Vespertilionidae* | (Garnham et al. 1971; D’Auria et al. 2010; Cicuttin et al. 2013, 2017; Reeves et al. 2016; Dietrich et al. 2016, 2017; Leulmi et al. 2016; Moreira-Soto et al. 2017; Vengust et al. 2018; Han et al. 2018b; Hornok et al. 2018; Szubert-Kruszyńska et al. 2019; Müller et al. 2020; Zhao et al. 2020; Matei et al. 2021; Selmi et al. 2021; Corduneanu et al. 2021; Mahyudin and Kumar 2022; Nawtaisong et al. 2022) | 20 |

**References**

Afonso E, Goydadin AC (2018) Molecular detection of *Anaplasma phagocytophilum* DNA in the lesser horseshoe bat (*Rhinolophus hipposideros*) guano. Epidemiol Infect 146:1253–1258. https://doi.org/10.1017/S0950268818001279

André MR, Gutiérrez R, Ikeda P, et al (2019) Genetic diversity of *Bartonella* spp. in vampire bats from Brazil. Transbound Emerg Dis 66:2329–2341. https://doi.org/10.1111/tbed.13290

Anh PH, Van Cuong N, Son NT, et al (2015) Diversity of *Bartonella* spp. in bats, southern Vietnam. Emerg Infect Dis 21:1266–1267. https://doi.org/10.3201/eid2107.141760

Bai Y, Kosoy M, Recuenco S, et al (2011) *Bartonella* spp. in bats, Guatemala. Emerg Infect Dis 17:1269–1272. https://doi.org/10.3201/eid1707.101867

Bai Y, Osinubi MOV, Osikowicz L, et al (2018) Human exposure to novel *Bartonella* species from contact with fruit bats. Emerg Infect Dis 24:2317–2323. https://doi.org/10.3201/eid2412.181204

Bai Y, Recuenco S, Gilbert AT, et al (2012) Prevalence and diversity of *Bartonella* spp. in bats in Peru. Am J Trop Med Hyg 87:518–523. https://doi.org/10.4269/ajtmh.2012.12-0097

Bai Y, Urushadze L, Osikowicz L, et al (2017) Molecular survey of bacterial zoonotic agents in bats from the country of Georgia (Caucasus). PLoS One 12:1–12. https://doi.org/10.1371/journal.pone.0171175

Ballados-González GG, Sánchez-Montes S, Romero-Salas D, et al (2018) Detection of pathogenic *Leptospira* species associated with phyllostomid bats (Mammalia: Chiroptera) from Veracruz, Mexico. Transbound Emerg Dis 65:773–781. https://doi.org/10.1111/tbed.12802

Banerjee A, Baid K, Byron T, et al (2020) Seroprevalence in bats and detection of *Borrelia burgdorferi* in bat ectoparasites. Microorganisms 8:440

Banskar S, Mourya DT, Shouche YS (2016) Bacterial diversity indicates dietary overlap among bats of different feeding habits. Microbiol Res 182:99–108. https://doi.org/10.1016/j.micres.2015.10.006

Becker DJ, Bergner LM, Bentz AB, et al (2018a) Genetic diversity, infection prevalence, and possible transmission routes of *Bartonella* spp. in vampire bats. PLoS Negl Trop Dis 12:1–21. https://doi.org/10.1371/journal.pntd.0006786

Becker DJ, Czirják G, Volokhov D V., et al (2018b) Livestock abundance predicts vampire bat demography, immune profiles and bacterial infection risk. Philos Trans R Soc B Biol Sci 373:. https://doi.org/10.1098/rstb.2017.0089

Becker DJ, Speer KA, Brown AM, et al (2020) Ecological and evolutionary drivers of haemoplasma infection and bacterial genotype sharing in a Neotropical bat community. Mol Ecol 29:1534–1549. https://doi.org/10.1111/mec.15422

Bessa TÁF, Spichler A, Berardis Chapola ÉG, et al (2010) The contribution of bats to leptospirosis transmission in São Paulo City, Brazil. Am J Trop Med Hyg 82:315–317. https://doi.org/10.4269/ajtmh.2010.09-0227

Bevans AI, Fitzpatrick DM, Stone DM, et al (2020) Phylogenetic relationships and diversity of bat-associated leptospira and the histopathological evaluation of these infections in bats from Grenada, West Indies. PLoS Negl Trop Dis 14:1–15. https://doi.org/10.1371/journal.pntd.0007940

Braga MD, Gonçalves L, Silva T, et al (2020) Occurrence of *Bartonella* genotypes in bats and associated Streblidae flies from Maranhão state, northeastern Brazil. Brazilian J Vet Parasitoloy 2961:1–7

Brook CE, Bai Y, Dobson AP, et al (2015) *Bartonella* spp. in fruit bats and blood-feeding ectoparasites in Madagascar. PLoS Negl Trop Dis 9:1–9. https://doi.org/10.1371/journal.pntd.0003532

Bunnell JE, Hice CL, Watts DM, et al (2000) Detection of pathogenic *Leptospira* spp. infections among mammals captured in the Peruvian Amazon basin region. Am J Trop Med Hyg 63:255–258. https://doi.org/10.4269/ajtmh.2000.63.255

Carvajal-Agudelo JD, Ramírez-Chaves HE, Ossa-López PA, Rivera-Páez FA (2022) Bacteria related to tick-borne pathogen assemblages in *Ornithodoros* cf. *hasei* (Acari: Argasidae) and blood of the wild mammal hosts in the Orinoquia region, Colombia. Exp Appl Acarol 87:253–271. https://doi.org/10.1007/s10493-022-00724-9

Cicuttin GL, Boeri EJ, Beltrán FJ, Dohmen FEG (2013) Molecular detection of *Neorickettsia risticii* in Brazilian free-tailed bats (*Tadarida brasiliensis*) from Buenos Aires, Argentina. Pesqui Vet Bras 33:648–650. https://doi.org/10.1590/S0100-736X2013000500016

Cicuttin GL, De Salvo MN, La Rosa I, Dohmen FEG (2017) *Neorickettsia risticii*, *Rickettsia* sp. and *Bartonella* sp. in *Tadarida brasiliensis* bats from Buenos Aires, Argentina. Comp Immunol Microbiol Infect Dis 52:1–5. https://doi.org/10.1016/j.cimid.2017.04.004

Collere FCM, Ferrari, L.D.R., Drozino, R.N., et al (2022) Hemotropic mycoplasmas in bats from forest fragments, state of Paraná, southern Brazil. Semin Ciências Agrárias 43:431–440. https://doi.org/10.5433/1679-0359.2022v43n1p431

Colunga-Salas P, Sánchez-Montes S, León-Paniagua L, Becker I (2020) *Borrelia* in neotropical bats: Detection of two new phylogenetic lineages. Ticks Tick Borne Dis 12:. https://doi.org/10.1016/j.ttbdis.2020.101642

Concannon R, Wynn-Owen K, Simpson VR, Birtles RJ (2005) Molecular characterization of haemoparasites infecting bats (Microchiroptera) in Cornwall, UK. Parasitology 131:489–496. https://doi.org/10.1017/S0031182005008097

Corduneanu A, Mihalca AD, Sándor AD, et al (2021) The heart microbiome of insectivorous bats from Central and South Eastern Europe. Comp Immunol Microbiol Infect Dis 75:. https://doi.org/10.1016/j.cimid.2020.101605

Corduneanu A, Sándor AD, Ionicǎ AM, et al (2018) *Bartonella* DNA in heart tissues of bats in central and eastern Europe and a review of phylogenetic relations of bat-associated bartonellae. Parasites and Vectors 11:1–7. https://doi.org/10.1186/s13071-018-3070-7

Correia dos Santos L, Vidotto O, dos Santos NJR, et al (2020) Hemotropic mycoplasmas (hemoplasmas) in free-ranging bats from Southern Brazil. Comp Immunol Microbiol Infect Dis 69:101416. https://doi.org/10.1016/j.cimid.2020.101416

Cox TE, Smythe LD, Leung LKP (2005) Flying foxes as carriers of pathogenic *Leptospira* species. J Wildl Dis 41:753–757. https://doi.org/10.7589/0090-3558-41.4.753

D’Auria SRN, Camargo MCGO, Pacheco RC, et al (2010) Serologic survey for rickettsiosis in bats from São Paulo City, Brazil. Vector-Borne Zoonotic Dis 10:459–463. https://doi.org/10.1089/vbz.2009.0070

Davoust B, Marié J-L, Dahmani M, et al (2016) Evidence of *Bartonella* spp. in blood and ticks (*Ornithodoros hasei*) of bats, in French Guiana. Vector-Borne Zoonotic Dis 16:516–519. https://doi.org/10.1089/vbz.2015.1918

Davoust B, Marié JL, de Santi VP, et al (2014) Three-toed sloth as putative reservoir of *Coxiella burnetii*, Cayenne, French Guiana. Emerg Infect Dis 20:1960

Descloux E, Mediannikov O, Gourinat A, et al (2021) Flying fox haemolytic fever, description of a new zoonosis caused by “*Candidatus* Mycoplasma haemohominis.” Clin Infect Dis ciaa1648:1–11

Desvars A, Naze F, Benneveau A, et al (2013) Endemicity of leptospirosis in domestic and wild animal species from Reunion Island (Indian Ocean). Epidemiol Infect 141:1154–1165. https://doi.org/10.1017/S0950268812002075

Desvars A, Naze F, Vourc’h G, et al (2012) Similarities in *Leptospira* serogroup and species distribution in animals and humans in the Indian ocean island of Mayotte. Am J Trop Med Hyg 87:134–140. https://doi.org/10.4269/ajtmh.2012.12-0102

Di Cataldo S, Kamani J, Cevidanes A, et al (2020) Hemotropic mycoplasmas in bats captured near human settlements in Nigeria. Comp Immunol Microbiol Infect Dis 70:101448. https://doi.org/10.1016/j.cimid.2020.101448

Dietrich M, Gomard Y, Lagadec E, et al (2018a) Biogeography of *Leptospira* in wild animal communities inhabiting the insular ecosystem of the western Indian Ocean islands and neighboring Africa article. Emerg Microbes Infect 7:1–12. https://doi.org/10.1038/s41426-018-0059-4

Dietrich M, Kearney T, Seamark ECJ, et al (2018b) Synchronized shift of oral, faecal and urinary microbiotas in bats and natural infection dynamics during seasonal reproduction. R Soc Open Sci 5:. https://doi.org/10.1098/rsos.180041

Dietrich M, Kearney T, Seamark ECJ, Markotter W (2017) The excreted microbiota of bats: Evidence of niche specialisation based on multiple body habitats. FEMS Microbiol Lett 364:1–7. https://doi.org/10.1093/femsle/fnw284

Dietrich M, Tjale MA, Weyer J, et al (2016) Diversity of *Bartonella* and *Rickettsia* spp. in bats and their blood-feeding ectoparasites from South Africa and Swaziland. PLoS One 11:1–9. https://doi.org/10.1371/journal.pone.0152077

Dietrich M, Wilkinson DA, Benlali A, et al (2015) *Leptospira* and paramyxovirus infection dynamics in a bat maternity enlightens pathogen maintenance in wildlife. Environ Microbiol 17:4280–4289. https://doi.org/10.1111/1462-2920.12766

Dietrich M, Wilkinson DA, Soarimalala V, et al (2014) Diversification of an emerging pathogen in a biodiversity hotspot: *Leptospira* in endemic small mammals of Madagascar. Mol Ecol 23:2783–2796. https://doi.org/10.1111/mec.12777

Emanuel ML, Mackerras IM, Smith DJW (1964) The epidemiology of leptospirosis in North Queensland: I. General survey of animal hosts. Epidemiol Infect 62:451–484. https://doi.org/10.1017/S0022172400040195

Evans NJ, Bown K, Timofte D, et al (2009) Fatal borreliosis in bat caused by relapsing fever spirochete, United Kingdom. Emerg Infect Dis 15:1331–1333. https://doi.org/10.3201/eid1508.090475

Everard CO, Fraser-Chanpong GM, Bhagwandin LJ, et al (1983) Leptospires in wildlife from Trinidad and Grenada. J Wildl Dis 19:192–199. https://doi.org/10.7589/0090-3558-19.3.192

Fennestad KL, Borg-Petersen C (1972) Leptospirosis in Danish wild mammals. J Wildl Dis 8:343–351. https://doi.org/10.7589/0090-3558-8.4.343

Ferreira ACR, Vieira TM, da Costa Custódio DA, et al (2021) Cross-sectional study on *Brucella* spp., *Leptospira* spp. and *Salmonella* spp. in bats from Montes Claros, Minas Gerais, Brazil. Comp Immunol Microbiol Infect Dis 78:101692. https://doi.org/10.1016/j.cimid.2021.101692

Ferreira dos Santos LG (2013) Absence of Anaplasmataceae DNA in wild birds and bats from a flooded area in the brazilian northern pantanal. Air Water Borne Dis 02:1–5. https://doi.org/10.4172/2167-7719.1000113

Ferreira MS, Guterres A, Rozental T, et al (2018) *Coxiella* and *Bartonella* spp. in bats (Chiroptera) captured in the Brazilian Atlantic Forest biome. BMC Vet Res 14:1–10. https://doi.org/10.1186/s12917-018-1603-0

Fritschi J, Fritschi J, Marti H, et al (2020) Prevalence and phylogeny of Chlamydiae and hemotropic mycoplasma species in captive and free-living bats. BMC Microbiol 20:1–32. https://doi.org/10.1186/s12866-020-01872-x

Gardner RA, Molyneux DH, Stebbings RE (1987) Studies on the prevalence of haematozoa of British bats. Mamm Rev 17:75–80. https://doi.org/10.1111/j.1365-2907.1987.tb00051.x

Gardon J, He J, Fouquet E, et al (2001) Suburban transmission of Q fever in French Guiana: Evidence of a wild reservoir. J Infect Dis 184:278–284

Garnham PCC, Lainson R, Shaw JJ (1971) A contribution to the study of the *Haematozoon* parasites of bats: A new mammalian haemoproteid, *Polychromophilus deanei* n. sp. Mem Inst Oswaldo Cruz 69:119–125

Gibson KE, Rikihisa Y, Zhang C, Martin C (2005) *Neorickettsia risticii* is vertically transmitted in the trematode *Acanthatrium oregonense* and horizontally transmitted to bats. Environ Microbiol 7:203–212. https://doi.org/10.1111/j.1462-2920.2004.00683.x

Goedbloed E, Cremers-Hoyer L, Perié NM (1964) Blood parasites of bats in the Netherlands. Ann Trop Med Parasitol 58:257–260. https://doi.org/10.1080/00034983.1964.11686240

Gomard Y, Dietrich M, Wieseke N, et al (2016) Malagasy bats shelter a considerable genetic diversity of pathogenic *Leptospira* suggesting notable host-specificity patterns. FEMS Microbiol Ecol 92:1–12. https://doi.org/10.1093/femsec/fiw037

Gonçalves-Oliveira J, Rozental T, Guterres A, et al (2020) Investigation of *Bartonella* spp. in Brazilian mammals with emphasis on rodents and bats from the Atlantic Forest. Int J Parasitol Parasites Wildl 13:80–89. https://doi.org/10.1016/j.ijppaw.2020.07.004

Han H-J, Liu J-W, Wen H-L, et al (2020) Pathogenic new world relapsing fever *Borrelia* in a *Myotis* bat, Eastern China, 2015 . Emerg Infect Dis 26:3083–3085. https://doi.org/10.3201/eid2612.191450

Han H-J, Wen H, Liu J, et al (2018a) Pathogenic *Leptospira* species in insectivorous bats, China, 2015. Emerg Infect Dis 24:1123–1127

Han HJ, Li ZM, Li X, et al (2021) Bats and their ectoparasites (Nycteribiidae and Spinturnicidae) carry diverse novel *Bartonella* genotypes, China. Transbound Emerg Dis 1–14. https://doi.org/10.1111/tbed.14357

Han HJ, Liu JW, Wen HL, et al (2018b) *Babesia vesperuginis* in insectivorous bats from China. Parasites and Vectors 11:1–5. https://doi.org/10.1186/s13071-018-2902-9

Han HJ, Wen HL, Zhao L, et al (2017) Novel *Bartonella* species in insectivorous bats, Northern China. PLoS One 12:1–9. https://doi.org/10.1371/journal.pone.0167915

Hanson AW (1970) Isolation of spirochaetes from primates and other mammalian species. Br J Vener Dis 49:303–306

Harkin KR, Hays M, Davis R, Moore M (2014) Use of PCR to identify *Leptospira* in kidneys of big brown bats (*Eptesicus fuscus*) in Kansas and Nebraska, USA. J Wildl Dis 50:651–654. https://doi.org/10.7589/2013-08-201

Holz PH, Lumsden LF, Legione AR, Hufschmid J (2019) *Polychromophilus melanipherus* and haemoplasma infections not associated with clinical signs in southern bent-winged bats (*Miniopterus orianae bassanii*) and eastern bent-winged bats (*Miniopterus orianae oceanensis*). Int J Parasitol Parasites Wildl 8:10–18. https://doi.org/10.1016/j.ijppaw.2018.11.008

Hornok S, Szőke K, Estók P, et al (2018) Assessing bat droppings and predatory bird pellets for vector-borne bacteria: molecular evidence of bat-associated *Neorickettsia* sp. in Europe. Antonie van Leeuwenhoek, Int J Gen Mol Microbiol 111:1707–1717. https://doi.org/10.1007/s10482-018-1043-7

Hou SL, Koh FX, Idris N, et al (2018) Molecular detection of *Bartonella* spp. in Malaysian small flying foxes (*Pteropus hypomelanus*). Trop Biomed 35:293–299

Ikeda P, Seki MC, Carrasco AOT, et al (2017) Evidence and molecular characterization of *Bartonella* spp. and hemoplasmas in neotropical bats in Brazil. Epidemiol Infect 145:2038–2052. https://doi.org/10.1017/S0950268817000966

Ikeda P, Torres JM, Lourenço EC, et al (2022) Molecular detection and genotype diversity of hemoplasmas in non-hematophagous bats and associated ectoparasites sampled in peri-urban areas from Brazil. Acta Trop 225:. https://doi.org/10.1016/j.actatropica.2021.106203

Ikeda P, Torres JM, Perles L, et al (2020) Intra-and inter-host assessment of *Bartonella* diversity with focus on non-hematophagous bats and associated ectoparasites from Brazil. Microorganisms 8:1–20. https://doi.org/10.3390/microorganisms8111822

Ikeda P, Torres JM, Placa AJV, et al (2021) Molecular survey of Anaplasmataceae agents and Coxiellaceae in non-hematophagous bats and associated ectoparasites from Brazil. Parasitologia 1:197–209. https://doi.org/10.3390/parasitologia1040021

Imnadze T, Natradze I, Zhgenti E, et al (2020) Identification of a novel *Yersinia enterocolitica* strain from bats in association with a bat die-off that occurred in Georgia (Caucasus). Microorganisms 8:1–11. https://doi.org/10.3390/microorganisms8071000

Judson SD, Frank HK, Hadly EA (2015) Bartonellae are prevalent and diverse in Costa Rican bats and bat flies. Zoonoses Public Health 62:609–617. https://doi.org/10.1111/zph.12188

Kamani J, Baneth G, Mitchell M, et al (2014) *Bartonella* species in bats (Chiroptera) and bat flies (Nycteribiidae) from Nigeria, West Africa. Vector-Borne Zoonotic Dis 14:625–632. https://doi.org/10.1089/vbz.2013.1541

Kamani J, Harrus S, Ocholi RA, et al (2021) Molecular detection and characterization of pathogenic *Leptospira* species in bats (Chiroptera) roosting in human habitats in Nigeria, West Africa. Zoonoses Public Health 68:908–916. https://doi.org/10.1111/zph.12880

Kosoy M, Bai Y, Lynch T, et al (2010) *Bartonella* spp. in bats, Kenya. Emerg Infect Dis 16:1875–1881. https://doi.org/10.3201/eid1612.100601

Lagadec E, Gomard Y, Guernier V, et al (2012) Pathogenic *Leptospira* spp. in bats, Madagascar and Union of the Comoros. Emerg Infect Dis 18:1696–1698. https://doi.org/10.3201/eid1810.111898

Lagadec E, Gomard Y, Le Minter G, et al (2016) Identification of *Tenrec ecaudatus*, a wild mammal introduced to Mayotte Island, as a reservoir of the newly identified human pathogenic *Leptospira mayottensis*. PLoS Negl Trop Dis 10:1–12. https://doi.org/10.1371/journal.pntd.0004933

Leulmi H, Aouadi A, Bitam I, et al (2016) Detection of *Bartonella tamiae*, *Coxiella burnetii* and rickettsiae in arthropods and tissues from wild and domestic animals in northeastern Algeria. Parasites and Vectors 9:1–8. https://doi.org/10.1186/s13071-016-1316-9

Li D, Yang W, Li Q, et al (2021a) High prevalence and genetic variation of *Bartonella* species inhabiting the bats in southwestern Yunnan. Biodivers Sci 29:1245–1255. https://doi.org/10.17520/biods.

Li ZM, Xiao X, Zhou CM, et al (2021b) Human-pathogenic relapsing fever *Borrelia* found in bats from central China phylogenetically clustered together with relapsing fever borreliae reported in the new world. PLoS Negl Trop Dis 15:1–11. https://doi.org/10.1371/journal.pntd.0009113

Lilley TM, Veikkolainen V, Pulliainen AT (2015) Molecular detection of *Candidatus* Bartonella hemsundetiensis in bats. Vector-Borne Zoonotic Dis 15:706–708. https://doi.org/10.1089/vbz.2015.1783

Lilley TM, Wilson CA, Bernard RF, et al (2017) Molecular detection of *Candidatus* Bartonella mayotimonensis in North American Bats. Vector-Borne Zoonotic Dis 17:243–246. https://doi.org/10.1089/vbz.2016.2080

Lin JW, Hsu YM, Chomel BB, et al (2012) Identification of novel *Bartonella* spp. in bats and evidence of Asian gray shrew as a new potential reservoir of *Bartonella*. Vet Microbiol 156:119–126. https://doi.org/10.1016/j.vetmic.2011.09.031

Lins ZC, Rosa CAS (1976) Investigações epidemiológicas preliminares sobre leptospiroses em Humboldt, Aripuanã, Mato Grosso. Acta Amaz 6:49–53. https://doi.org/10.1590/1809-43921976064s049

Mahyudin A, Kumar VS (2022) Detection of viral and bacterial pathogens in the population of bats from the east coast regions of Sabah, Malaysia. Short Commun Biotechnol 7:1-12.

Marinkelle CJ, Grose ES (1968) Species of *Borrelia* from a Colombian bat (*Natalus tumidirostris*). Nature 218:487–487

Mascarelli PE, Keel MK, Yabsley M, et al (2014) Hemotropic mycoplasmas in little brown bats (*Myotis lucifugus*). Parasites and Vectors 7:23–27. https://doi.org/10.1186/1756-3305-7-117

Matei IA, Corduneanu A, Sándor AD, et al (2021) *Rickettsia* spp. in bats of Romania: high prevalence of *Rickettsia monacensis* in two insectivorous bat species. Parasites and Vectors 14:1–9. https://doi.org/10.1186/s13071-021-04592-x

Mateus J, Gómez N, Herrera-Sepúlveda MT, et al (2019) Bats are a potential reservoir of pathogenic leptospira species in Colombia. J Infect Dev Ctries 13:278–283. https://doi.org/10.3855/jidc.10642

Matthias MA, Díaz MM, Campos KJ, et al (2005) Diversity of bat-associated *Leptospira* in the Peruvian Amazon inferred by Bayesian phylogenetic analysis of 16s ribosomal DNA sequences. Am J Trop Med Hyg 73:964–974. https://doi.org/10.4269/ajtmh.2005.73.964

Mayer FQ, Dos Reis EM, Bezerra AVA, et al (2017) Pathogenic *Leptospira* spp. in bats: Molecular investigation in Southern Brazil. Comp Immunol Microbiol Infect Dis 52:14–18. https://doi.org/10.1016/j.cimid.2017.05.003

McKee CD, Bai Y, Webb CT, Kosoy MY (2021) Bats are key hosts in the radiation of mammal-associated *Bartonella* bacteria. Infect Genet Evol 89:104719. https://doi.org/10.1016/j.meegid.2021.104719

McKee CD, Kosoy MY, Bai Y, et al (2017) Diversity and phylogenetic relationships among *Bartonella* strains from Thai bats. PLoS One 12:1–19. https://doi.org/10.1371/journal.pone.0181696

Mgode GF, Mbugi HA, Mhamphi GG, et al (2014) Seroprevalence of *Leptospira* infection in bats roosting in human settlements in Morogoro municipality in Tanzania. Tanzan J Health Res 16:1–7. https://doi.org/10.4314/thrb.v16i1.4

Millán J, Cevidanes A, Sacristán I, et al (2019) Detection and characterization of hemotropic mycoplasmas in bats in Chile. J Wildl Dis 55:977–981. https://doi.org/10.7589/2018-12-290

Millán J, López-Roig M, Delicado V, et al (2015) Widespread infection with hemotropic mycoplasmas in bats in Spain, including a hemoplasma closely related to “*Candidatus* Mycoplasma hemohominis.” Comp Immunol Microbiol Infect Dis 39:9–12. https://doi.org/10.1016/j.cimid.2015.01.002

Mitchell MM, Vicente-Santos, A. Rodríguez-Herrera, B. Corrales-Aguilar E, Gillespie TR (2022) Genetic diversity of *Bartonella* spp. in cave dwelling bats and bat flies, Costa Rica, 2018. Emerg Infect Dis 28:488

Monroy FP, Solari S, Lopez JÁ, et al (2021) High diversity of *Leptospira* species infecting bats captured in the Urabá region (Antioquia-colombia). Microorganisms 9:. https://doi.org/10.3390/microorganisms9091897

Moreira-Soto RD, Moreira-Soto A, Corrales-Aguilar E, et al (2017) ‘*Candidatus* Rickettsia nicoyana’: A novel *Rickettsia* species isolated from *Ornithodoros knoxjonesi* in Costa Rica. Ticks Tick Borne Dis 8:532–536. https://doi.org/10.1016/j.ttbdis.2017.02.015

Müller A, Sepúlveda P, Di Cataldo S, et al (2020) Molecular investigation of zoonotic intracellular bacteria in Chilean bats. Comp Immunol Microbiol Infect Dis 73:1–7. https://doi.org/10.1016/j.cimid.2020.101541

Muñoz-Leal S, Faccini-Martínez ÁA, Pérez-Torres J, et al (2020) Novel *Borrelia* genotypes in bats from the Macaregua Cave, Colombia. Zoonoses Public Health 1–7. https://doi.org/10.1111/zph.12789

Nabeshima K, Sato S, Brinkerhoff RJ, et al (2022) Prevalence and genetic diversity of *Bartonella* spp. in northern bats (*Eptesicus nilssonii*) and their blood-sucking ectoparasites in Hokkaido, Japan. Microb Ecol. https://doi.org/10.1007/s00248-021-01935-0

Nabeshima K, Sato S, Kabeya H, et al (2020) Isolation and genetic properties of *Bartonella* in eastern bent-wing bats (*Miniopterus fuliginosus*) in Japan. Infect Genet Evol 83:104354. https://doi.org/10.1016/j.meegid.2020.104354

Nawtaisong P, Robinson MT, Khammavong K, et al (2022) Zoonotic pathogens in wildlife traded in markets for human consumption, Laos. Emerg Infect Dis 28:860–864. https://doi.org/10.3201/eid2804.210249

Newman MM, Kloepper LN, Duncan M, et al (2018) Variation in bat guano bacterial community composition with depth. Front Microbiol 9:1–9. https://doi.org/10.3389/fmicb.2018.00914

Noguchi H (1919) Etiology of yellow fever: VIII. Presence of a *Leptospira* in wild animals in Guayaquil and its relation to *Leptospira icterohæmorrhagiæ* and *Leptospira icteroides*. J Exp Med 30:95–107

Ogawa H, Koizumi N, Ohnuma A, et al (2015) Molecular epidemiology of pathogenic *Leptospira* spp. in the straw-colored fruit bat (*Eidolon helvum*) migrating to Zambia from the Democratic Republic of Congo. Infect Genet Evol 32:143–147. https://doi.org/10.1016/j.meegid.2015.03.013

Olival KJ, Dittmar K, Bai Y, et al (2015) *Bartonella* spp. in a puerto rican bat community. J Wildl Dis 51:274–278. https://doi.org/10.7589/2014-04-113

Poofery J, Narapakdeesakul D, Riana E, et al (2021) Molecular identification and genetic diversity of *Bartonella* spp. in 24 bat species from Thailand. Transbound Emerg Dis 69: e717-e733

Pusterla N, Johnson EM, Chae JS, Madigan JE (2003) Digenetic trematodes, *Acanthatrium* sp. and *Lecithodendrium* sp., as vectors of *Neorickettsia risticii*, the agent of Potomac horse fever . J Helminthol 77:335–339. https://doi.org/10.1079/joh2003181

Qiu Y, Kajihara M, Nakao R, et al (2020) Isolation of *Candidatus* Bartonella rousetti and other bat-associated bartonellae from bats and their flies in Zambia. Pathogens 9:1–14. https://doi.org/10.3390/pathogens9060469

Qiu Y, Nakao R, Hangombe BM, et al (2019) Human borreliosis caused by a new world relapsing fever borrelia-like organism in the old world. Clin Infect Dis 69:107–112. https://doi.org/10.1093/cid/ciy850

Ramirez NN, Alegre EA, Ruiz RM, et al (2014) Detección de leptospiras patógenas en tejido renal de murciélagos de Corrientes, Argentina. Rev Vet 25:16–20. https://doi.org/10.30972/vet.251543

Ramos-Nino ME, Fitzpatrick DM, Eckstrom KM, et al (2021) The kidney-associated microbiome of wild-caught *Artibeus* spp. in Grenada, West Indies. Animals 11:1–8

Raya AP, Jaffe DA, Chomel BB, et al (2018) Detection of Bartonella species, including *Candidatus* Bartonella ovis sp. nov, in ruminants from Mexico and lack of evidence of *Bartonella* DNA in saliva of common vampire bats (Desmodus rotundus) predating on them. Vet Microbiol 222:69–74. https://doi.org/10.1016/j.vetmic.2018.06.018

Reeves WK, Beck J, Orlova M V., et al (2016) Ecology of bats, their ectoparasites, and associated pathogens on Saint Kitts Island. J Med Entomol 53:1218–1225. https://doi.org/10.1093/jme/tjw078

Reeves WK, Streicker DG, Loftis AD, Dasch GA (2006) Serologic survey of *Eptesicus fuscus* from Georgia, U.S.A. for *Rickettsia* and *Borrelia* and laboratory transmission of a *Rickettsia* by bat ticks. J Vector Ecol 31:386–389. https://doi.org/10.3376/1081-1710(2006)31[386:ssoeff]2.0.co;2

Ricciardi A, Ilvan D, Marly P, et al (1976) Anti-*Brucella* agglutinins in bats and “caluthrix” monkeys. J Wildl Dis 12:52–54

Sanseverino L, Filho HO, Esteve-Gassent M, Jorge TMR (2019) Test for *Borrelia* spp. in bats in an urban area in the South of Brazil. Rev Soc Bras Med Trop 52:0–3. https://doi.org/10.1590/0037-8682-0234-2019

Saraullo V, Loffler SG, Pastorino F, et al (2021) First report of pathogenic *Leptospira* spp. in Tadarida brasiliensis bats (family Molossidae) and *Eptesicus furinalis* (family Vespertilionidae) of Argentina. New host species in this country? Rev Argent Microbiol 53:210–215. https://doi.org/10.1016/j.ram.2020.09.007

Sebek Z (1975) Blood parasites of small wild mammals in Czechoslovakia. Folia Parasitol (Praha) 22:11–20

Sebek Z, Sixl W, Reinthaler F, et al (1989) Results of serological examination for leptospirosis of domestic and wild animals in the Upper Nile province (Sudan). J Hyg Epidemiol Microbiol Immunol 33:337–345

Seidlova V, Nemcova M, Pikula J, et al (2021) Urinary shedding of leptospires in palearctic bats. Transbound Emerg Dis 68:3089–3095. https://doi.org/10.1111/tbed.14011

Seidlova V, Straková P, Kejíková R, et al (2022) Detection of *Leptospira* species in bat cadavers, Czech and Slovak Republics . Emerg Microbes Infect 1–12. https://doi.org/10.1080/22221751.2022.2117095

Selmi R, Belkahia H, Dhibi M, et al (2021) First case of *Ehrlichia canis* infection in the common pipistrelle bat (*Pipistrellus pipistrellus*). Vet Rec Case Reports 9:190

Silva-Ramos CR, Chala-Quintero SM, Faccini-Martínez ÁA, et al (2022a) Pathogenic *Leptospira* species in bats: Molecular detection in a colombian cave. Trop Med Infect Dis 7:84. https://doi.org/10.3390/tropicalmed7060084

Silva-Ramos CR, Faccini-Martínez ÁA, Pérez-Torres J, et al (2022b) First molecular evidence of *Coxiella burnetii* in bats from Colombia. Res Vet Sci 150:33–35. https://doi.org/10.1016/j.rvsc.2022.05.009

Smythe LD, Field HE, Barnett LJ, et al (2002) Leptospiral antibodies in flying foxes in Australia. J Wildl Dis 38:182–186. https://doi.org/10.7589/0090-3558-38.1.182

Soupé‐Gilbert ME, Oedin M, Kainiu M, et al (2022) Original *Leptospira* spp. in island’s native terrestrial mammals: A case study in *Pteropus* spp. bats of New Caledonia. Transbound Emerg Dis Accepted:

Stuckey MJ, Boulouis H, Cliquet F, et al (2017a) Potentially zoonotic *Bartonella* in bats from France and Spain. Emerg Infect Dis 23:539–541

Stuckey MJ, Chomel BB, Galvez-Romero G, et al (2017b) *Bartonella* infection in hematophagous, insectivorous, and phytophagous bat populations of Central Mexico and the Yucatan Peninsula. Am J Trop Med Hyg 97:413–422. https://doi.org/10.4269/ajtmh.16-0680

Sun DL, Gao YZ, Ge XY, et al (2020) Special features of bat microbiota differ from those of terrestrial mammals. Front Microbiol 11:1–12. https://doi.org/10.3389/fmicb.2020.01040

Szentiványi T, Markotter W, Dietrich M, et al (2020) Host conservation through their parasites: Molecular surveillance of vector-borne microorganisms in bats using ectoparasitic bat flies. Parasite 27:1–10. https://doi.org/10.1051/parasite/2020069

Szubert-Kruszyńska A, Stańczak J, Cieniuch S, et al (2019) *Bartonella* and *Rickettsia* infections in haematophagous *Spinturnix myoti* mites (Acari: Mesostigmata) and their bat host, *Myotis myotis* (Yangochiroptera: Vespertilionidae), from Poland. Microb. Ecol. 77:759–768

Tagi-Zade TA, Mardanly AS, Akhmedov IB, et al (1970) Examination of bats for leptospirosis in the territory of Azerbaijan SSR. Zhurnal Mikrobiol Epidemiol i Immunobiol 9:118–121

Thayaparan S, Robertson IAN, Amraan F, et al (2013) Serological prevalence of leptospiral infection in wildlife in Sarawak, Malaysia. Borneo J Resour Sci Tech 2:71–74

Thayaparan S, Robertson ID, Abdullah MT (2015) Serological and molecular detection of *Leptospira* spp. from small wild mammals captured in Sarawak, Malaysia. Malays J Microbiol 11:93–101. https://doi.org/10.21161/mjm.67514

Torres-Castro M, Febles-Solís V, Hernández-Betancourt S, et al (2020) Pathogenic *Leptospira* in bats from Campeche and Yucatán, Mexico. J MVZ Cordoba 25:e1815

Tozer SJ, Lambert SB, Strong CL, et al (2014) Potential animal and environmental sources of Q Fever infection for humans in Queensland. Zoonoses Public Health 61:105–112. https://doi.org/10.1111/zph.12051

Tulsiani SM, Graham GC, Dohnt MF, et al (2011) Maximizing the chances of detecting pathogenic leptospires in mammals: The evaluation of field samples and a multi-sample-per-mammal, multi-test approach. Ann Trop Med Parasitol 105:145–162. https://doi.org/10.1179/136485911X12899838683205

Urushadze L, Bai Y, Osikowicz L, et al (2017) Prevalence, diversity, and host associations of *Bartonella* strains in bats from Georgia (Caucasus). PLoS Negl Trop Dis 11:1–19. https://doi.org/10.1371/journal.pntd.0005428

Veikkolainen V, Vesterinen EJ, Lilley TM, Pulliainen AT (2014) Bats as reservoir hosts of human bacterial pathogen, *Bartonella mayotimonensis*. Emerg Infect Dis 20:960–967. https://doi.org/10.3201/eid2006.130956

Vengust M, Knapic T, Weese JS (2018) The fecal bacterial microbiota of bats; Slovenia. PLoS One 13:1–15. https://doi.org/10.1371/journal.pone.0196728

Victoria RJ, Iriarte LJ, Sampedro AC (2018) Presence of leptospire spp. in urban bats from Sincelejo, Sucre, Colombia. Int J PharmTech Res 11:218–225. https://doi.org/10.20902/ijptr.2018.11303

Volokhov D V., Becker DJ, Bergner LM, et al (2017) Novel hemotropic mycoplasmas are widespread and genetically diverse in vampire bats. Epidemiol Infect 145:1–14. https://doi.org/10.1017/S095026881700231X

Wray AK, Olival KJ, Morán D, et al (2016) Viral diversity, prey preference, and *Bartonella* prevalence in Desmodus rotundus in Guatemala. Ecohealth 13:761–774. https://doi.org/10.1007/s10393-016-1183-z

Yadav MP, Sethi MS (1980) A study on the reservoir status of Q-fever in avifauna, wild mammals and poikilotherms in Uttar Pradesh (India). Int J Zoonoses 7:85–89

Yuan Z, Yu Y, Wang Y, et al (2019) Microbial diversity in the gastrointestinal tract of a bat, *Hypsugo alaschanicus*. Pak J Zool 51:1807–1813. https://doi.org/10.17582/journal.pjz/2019.51.5.1807.1813

Zabashta M V., Orlova M V., Pichurina NL, et al (2019) Participation of bats (Chiroptera, Mammalia) and their ectoparasites in circulation of pathogens of natural focal infections in the south of Russia. Entomol Rev 99:513–521. https://doi.org/10.1134/S0013873819040110

Zetun CB, Hoffmann JL, Silva RC, et al (2009) *Leptospira* spp. and *Toxoplasma gondii* antibodies in vampire bats (*Desmodus rotundus*) in Botucatu region, SP, Brazil. J Venom Anim Toxins Incl Trop Dis 15:546–552

Zhao M, Xiao X, Han HJ, et al (2019) *Leptospira* in bats from Hubei Province, China, 2018. J Wildl Dis 55:940–943. https://doi.org/10.7589/2019-01-009

Zhao S, Yang M, Liu G, et al (2020) Rickettsiae in the common pipistrelle *Pipistrellus pipistrellus* (Chiroptera: Vespertilionidae) and the bat soft tick *Argas vespertilionis* (Ixodida: Argasidae). Parasites and Vectors 13:4–9. https://doi.org/10.1186/s13071-020-3885-x
